# Nrf2/Keap1/ARE regulation by plant secondary metabolites: a new horizon in brain tumor management

**DOI:** 10.1186/s12964-024-01878-2

**Published:** 2024-10-15

**Authors:** Saikat Dewanjee, Hiranmoy Bhattacharya, Chiranjib Bhattacharyya, Pratik Chakraborty, Joshua Fleishman, Athanasios Alexiou, Marios Papadakis, Saurabh Kumar Jha

**Affiliations:** 1 Advanced Pharmacognosy Research Laboratory, Department of Pharmaceutical Technology, Jadavpur University, Kolkata, 700032 West Bengal India; 2 Department of Pharmaceutical Sciences, College of Pharmacy and Health Sciences, St. John’s University, New York, NY 11439 USA; 3 University Centre for Research & Development, Chandigarh University, Chandigarh-Ludhiana Highway, Mohali, Punjab India; 4 Department of Research & Development, Funogen, Athens, 11741 Greece; 5 Department of Research & Development, AFNP Med, Wien, 1030 Austria; 6 Department of Science and Engineering, Novel Global Community Educational Foundation, Hebersham, NSW 2770 Australia; 7 Department of Surgery II, University Hospital Witten-Herdecke, University of Witten-Herdecke, Heusnerstrasse 40, 42283 Wuppertal, Germany; 8 Department of Zoology, Kalindi College, University of Delhi, Delhi, 110008 India

**Keywords:** Antioxidants, ARE, Brain cancer, Keap1, Nrf2, Oxidative stress, Secondary metabolites

## Abstract

Brain cancer is regarded as one of the most life-threatening forms of cancer worldwide. Oxidative stress acts to derange normal brain homeostasis, thus is involved in carcinogenesis in brain. The Nrf2/Keap1/ARE pathway is an important signaling cascade responsible for the maintenance of redox homeostasis, and regulation of anti-inflammatory and anticancer activities by multiple downstream pathways. Interestingly, Nrf2 plays a somewhat, contradictory role in cancers, including brain cancer. Nrf2 has traditionally been regarded as a tumor suppressor since its cytoprotective functions are considered to be the principle cellular defense mechanism against exogenous and endogenous insults, such as xenobiotics and oxidative stress. However, hyperactivation of the Nrf2 pathway supports the survival of normal as well as malignant cells, protecting them against oxidative stress, and therapeutic agents. Plants possess a pool of secondary metabolites with potential chemotherapeutic/chemopreventive actions. Modulation of Nrf2/ARE and downstream activities in a Keap1-dependant manner, with the aid of plant-derived secondary metabolites exhibits promise in the management of brain tumors. Current article highlights the effects of Nrf2/Keap1/ARE cascade on brain tumors, and the potential role of secondary metabolites regarding the management of the same.

## Introduction

Brain tumors are diverse group of primary or metastatic neoplasms developed in the central nervous system (CNS) from neuroglia or precursor cells. These tumors are markedly known for poor prognosis and low patient survival rates [1]. Brain cancer involves the growth of neoplastic cells originating from systemic neoplasms and subsequently spreading to the interior regions of brain parenchymal cells during the advanced stages of malignancy. Brain cancer patients only survive for about five years after disease onset, that too with a compromised quality of life [2]. Most of the modern-day therapies fail to treat brain cancer due to its distinctive microenvironmental features. Prevention of brain cancer is still deceiving scientists. It urges the need to opt for novel multi-target agents to bring the dysregulated mechanisms associated with cancer onset and progression back on track. Current research is concentrating on antioxidants since they can interfere with the onset and progression of tumors, including CNS tumors.

Nrf2/Keap1/ARE has been reported to orchestrate downstream oxidative mechanisms, which in turn regulate several inflammatory and apoptotic pathways [3]. The Nrf2/Keap1 pathway plays a crucial role in the regulation of genes responsible for antioxidant and cytoprotective functions. Nrf2, a transcription factor regulates the expression of multiple genes implicated in cellular defense against oxidative stress, a characteristic feature of various pathological conditions including tumors. The principal way to regulate Nrf2 activity is by interactions with the Keap1 protein. Under normoxic conditions, Keap1 binds to Nrf2, and directs it to proteosomal degradation, whilst Keap1 is regenerated. During oxidative stress the interactions between Nrf2 and Keap1 are interrupted, and Nrf2 activates the transcription of protective genes. Thus, activation of the Nrf2 cascade is considered a useful strategy to attenuate pathologies associated with oxidative stress. On the other hand, Nrf2 can also be regulated independent of Keap1, via β-TrCP pathway and BACH1 pathway. Upon phosphorylation of Nrf2 by GSK-3β, it becomes recognizable by β-TrCP. In turn, β-TrCP labels Nrf2 for ubiquitination regardless of whether it is mediated by engaging the Keap1/Cul3 complex or not [4]. In the nucleus, Nrf2 binds with Maf protein to form heterodimers, competitively displacing BACH1 from ARE-binding sites. This, in turn activates the antioxidant, anti-inflammatory and neuroprotective cascades. Clearly, downregulation of BACH1 would lead to activation of Nrf2-associated pathways [5].

The Nrf2 signaling cascade is considered a double-edged sword in that, to exert chemotherapeutic activities, Nrf2 needs to be downregulated while to prevent cancer recurrence, Nrf2 cascade needs to be upregulated. In this regard, plant secondary metabolites represent a great reservoir of potential chemotherapeutic/chemopreventive agents. Dose of the agent also plays a vital role to decide the response on Nrf2 cascade. Emerging evidence reveals that modulating Nrf2/ARE and downstream antioxidant enzymes in a Keap1-dependant manner, with the aid of multiple plant-derived secondary metabolites might play pivot in the management of brain tumors [6]. Present review highlights the effects of plant secondary metabolites via regulating Nrf2/Keap1/ARE, and downstream mediators in combating the pathogenesis of tumors in the brain.

## Epidemiology of brain tumors

Brain tumors are heterogeneous metastatic neoplastic developments in the CNS originating in neuroglia or precursor cells, characterized by poor prognosis and low patient survival rate. Primary brain tumors manifest within only a few months, while secondary brain tumors develop from primary tumors of lower grades. Patients with brain tumors typically survive for about five years after the commencement of the disease [7]. Brain tumors represent a complex etiology that may involve age, race, ethnicity, gender, environment, hormones, and heredity [8]. There exist more than 100 histologically distinct subtypes of brain tumor, prevalence depends mainly on age and tissue type. Glioblastoma is the most prevalent malignant brain tumor offering the highest fatality rate [9]. The occurrence of glioblastoma has nearly doubled in the last two decades. Certain tumors, such as pilocytic astrocytoma and subgroups of ependymomas, are more prevalent in children, while others, such as glioblastoma, are more common in the elderly [10]. Worldwide, about 3 per 100,000 persons suffer from some form of brain tumor yearly, with males dominating the list [11]. Brain cancer is estimated to feature among the top ten leading causes of cancer-related deaths globally in 2023 [12]. According to the statistics of the National Brain Tumor Society in 2016, approximately 78,000 people are diagnosed and 16,616 people die from malignant brain tumors in USA every year. Brain tumors are the most common form of cancer and the second highest cause of mortality in those under the age of 19 in both United States and Canada [9]. The incidence rate is estimated to reach 435,000 individuals with brain tumors by 2040 globally [13].

Factors including overexposure to ionizing radiation, ageing population, pollution, and stress can be attributed to the increase in the number of brain cancer patients. Treatment usually involves surgery followed by chemotherapy and radiation therapy, which suffers from limitations such as inappropriate delineation, inadequate delivery etc. High invasiveness of brain tumors, along with heterogeneity, is primarily accountable for the unsatisfactory performance of existing chemotherapies, the difficulties regarding total surgical excision, and the lowered radiation efficiency. This might pave the way to local recurrences. The unique microenvironmental and intrinsic cellular characteristics of brain tumors seem to make them practically resistant to majority of modern-day cutting-edge therapeutic modalities [14]. Several strategies have been developed against brain cancer, however only a few clinically approved drugs exist, thus leaving scope for novel treatment modalities. Even the existing drugs raise few concerns regarding safety, and toxicity at effective doses (Table 1). Contemporary research prioritizes the utilization of antioxidants in the chemoprevention and/or treatment of various forms of cancer including brain cancer, owing to their potential to impede carcinogenesis and minimize tumor growth [15].

**Table 1:** FDA-approved therapeutic strategies against brain cancer

Sl No.	Agents	Years of approval	Mechanisms	Uses	Points of concern
1	Lomustine	1976	Alkylating agent	High grade glioma	Hematologic toxicity
2	Carmustine	1977	Alkylating agent	Intracranial malignancies	Bone marrow suppression, ocular toxicity, pulmonary toxicity
3	Carmustine wafer implants	1996	Alkylating agent	Recurrent glioma	Intracranial infection, cerebral edema
4	Hypericin	2000	Downregulation of class I HDACs	Glioblastoma multiforme	Phototoxicity
5	Trabedersen	2002	Reduced production of TGF-β2	Malignant glioma	Thrombocytopenia
6	Erlotinib HCl	2003	Blockade of EGFRvIII	Malignant glioma	Ocular complications
7	Cilengitide	2005	Inhibition of integrins, FAK, SRC	Malignant glioma	Chance of intracranial haemorrhage
8	Enzastaurine	2005	Inhibitor of PI3K/Akt cascade	Glioblastoma multiforme	Alteration in QT-interval
9	Temozolamide	2005	Alkylating agent	High grade glioma	Hematologic toxicity
10	Procarbazine HCl	2006	Alkylating agent	Malignant glioma	Bone marrow complications
11	Bevacizumab	2009	VEGF-targeted antibody	Recurrent glioma	Throboembolic events, hypertension, cerebral bleeding
12	Cediranib	2010	Inhibition of angiogenesis	Glioblastoma	Hypertension
13	Optune device	2011	Tumor treating field	Glioma	Seizures
14	Afatinib	2014	Inhibition of EGFRvIII, FAK	Malignant tumors in CNS	Sensitive skin
15	Gamma tiles	2018	Radiation therapy	Recurrent brain tumors	Seizures, irritability

## Brain tumor pathomechanisms: crosstalk with oxidative stress

Brain comprises about 2% of the body, while it consumes nearly 20% of body’s oxygen, thus increasing the probability of free radical production compared to other organs. Oxidative stress arises from an imbalance between the production and build-up of reactive oxygen species (ROS) and reactive nitrogen species (RNS). Numerous studies revealed an association between oxidative stress and brain tumor development [11, 16]. At the physiological concentration, ROS regulates signal transduction, gene expression, enzyme activation, and protein folding [17]. Upon reaching the threshold levels of ROS, the body experiences a state of oxidative stress. During cerebral hypoxia, hypoxic areas promote tumorigenesis by increasing ROS concentrations in the brain. The increased levels of free radicals have been linked to oncogene activation, metabolism enhancement, and mitochondrial dysfunction [15]. The activation of hypoxia-inducible factors (HIFs) by ROS is believed to be involved in the development of tumors in the brain [18]. On the other hand, HIFs elicit ROS generation by means of activating NADPH oxidase [19]. In addition, HIFs also regulate apoptotic and cell cycle pathways by regulating numerous transcription factors. ROS stimulate lipid peroxidation, induce electron leakage, and interfere with calcium homeostasis [20]. The activation of protein kinases that stimulate cell proliferation is facilitated by the presence of intracellular free Ca^2+^ ions. ROS also induce NF-κB activation, which subsequently plays a crucial role in cellular proliferation, invasion, and metastasis [21]. Further, oxidative stress brings about free radical-induced alterations in the DNA, leading to genomic instability. Through epigenetic regulation, ROS induce histone deacetylases (HDACs) to activate oncogenes, and repress tumor suppressor genes [22]. Accumulation of free radicals decreases endogenous antioxidants too. The combination of genomic alterations in tissues, and reduction in cellular antioxidant levels represents correlation with carcinogenic and mutagenic outcomes.

Describing tumor gene expression patterns has become critical for developing clinically useful classifications and effective therapy options [23]. The World Health Organization’s current classification approach for glioblastoma is mainly based on mutation of isocitrate dehydrogenase [24]. Mutations in isocitrate dehydrogenase have been linked to elevated levels of HIF-1α and vascular endothelial growth factor (VEGF), which promote tumor progression and metastasis, while high levels of 2-hydroxyglutaric acid inhibit stem cell differentiation [25]. The literature reveals that Nrf2 plays a role in the development and progression of gliomas with isocitrate dehydrogenase mutations, further confirming an interplay between oxidative stress and isocitrate dehydrogenase mutations [26].

Emerging evidence suggests that a few products of lipid peroxidation, protein carbonylation, and DNA oxidation may serve as potential oxidative stress biomarkers in neurodegenerative diseases as well as in brain cancer [27–31]. Levels of these potential marker compounds provide an idea on status of certain oxidative stress-sensitive disorders including cancers. During the absence of symptoms of common neurodegenerative disorders, they hint at the possibility of onset or progress of brain tumors. Free radicals attack the unsaturated fatty acid moieties of membrane lipids, resulting in a self-replicating chain reaction of non-enzymatic lipid peroxidation that generates malondialdehyde, 4-hydroxynonenal, isoprostains, and other compounds capable to function as biomarkers of lipid peroxidation [32]. The extent of protein carbonylation can serve as redox marker for brain tumors. DNA oxidation has also been reported to be associated with cancer initiation whereby 8-hydroxy-2′- deoxyguanosine acts as a biomarker [33]. Human MutT homolog protein 1 (hMTH1), which catalyzes the hydrolysis of oxidized purine nucleoside triphosphates, is also a biomarker of oxidative DNA damage [34]. In addition, endogenous enzymatic antioxidants, such as superoxide dismutase (SOD), catalase (CAT), glutathione peroxidase (GPx), glutathione reductase, glutathione-S-transferase (GST) etc. and non-enzymatic antioxidants such as GSH may potentially act as oxidative stress biomarkers for brain tumors. Table 2 enlists the potential biomarkers of brain tumor, associated with oxidative stress.

**Table 2:** Oxidative stress related biomarkers, associated with brain tumor

Sl No.	Biomarker compounds	Types of biomarkers	Significance	References
1	4-hydroxynonenal	Product of lipid peroxidation	Degree of lipid peroxidation proportional to the extent of malignancy and neovascularization	[28, 29]
2	8-hydroxy-2′- deoxyguanosine	Product of DNA oxidation	Accumulation in high quantities in tumor tissues corresponds to high grade glioma	[27, 31]
3	GSH	Non-enzymatic antioxidant	Low GSH level hints at high susceptibility to oxidative stress and tumorigenesis	[32]
4	hMTH1	Product of DNA oxidation	High expression in tumor tissues corresponds to high grade glioma	[27]
5	Malondialdehyde	Product of lipid peroxidation	High levels in sera and tumor tissue indicate malignancy	[30]
6	Thiobarbituric acid reactive substances	Malondialdehyde equivalents	Elevated levels in peritumoral tissue corresponds to high grade intracranial tumors	[32]

Recent findings have shed light on the molecular mechanisms underlying the development of malignant brain tumors [16, 35]. Majority of primary glioblastomas exhibit amplification and activation of the epidermal growth factor receptor (EGFR). The EGFR variant III (EGFRvIII) has been observed to promote cellular proliferation and inhibit apoptosis through the suppression of p27 in a PI3K/Akt/mTOR-dependent manner [36]. The PI3K/Akt/mTOR signaling pathway is crucial in the advancement of tumors as it initiates angiogenesis, epithelial-mesenchymal transition (EMT), cell migration, and cell invasion, while also inhibiting apoptosis. The phosphatase and tensin homolog (PTEN) gene functions as a tumor suppressor by inhibiting cell proliferation through the formation of an inhibitory network on the PI3K/Akt signaling pathway. This is achieved by inducing the enzymatic activity of PIP3. Interestingly, mesenchymal glioblastoma is distinguished by the presence of genetic mutations affecting NF1 and PTEN, which subsequently lead to the activation of the MAPK and PI3K signaling pathways [37]. The upregulation of the EGFR gene is predominantly detected in cases of glioblastoma [38]. The initiation of secondary glioblastoma is attributed to the p53 pathway [39]. Apart from oxidative stress, progression of the disease is also significantly influenced by the tumor microenvironment.

## Nrf2/Keap1/ARE signaling: a double edged sword in tumor biology

Nrf2 has traditionally been considered to suppress tumor since its cytoprotective attributes are deemed to be the principal defense mechanism of cells against exogenous and endogenous insults, including xenobiotics and oxidative stress. However, several recent studies demonstrate that hyperactivation of the Nrf2 signaling cascade favors the survival of both normal and malignant cells, protecting them against oxidative stress, and chemotherapeutic agents [40, 41]. Hormetic Nrf2 modulators at low doses activate Nrf2 cascade [42]. Thus, activation of Nrf2 pathway in malignant cells might actually aid in progress of the existing disease. On the other hand, prevention and/or inhibition of Nrf2 pathway by high dose of hormetic Nrf2 modulators would lead to cell death by promoting ROS and doing away with chemoresistance in malignant cells [43]. Nrf2 does not function in normal oxygenated conditions. However, Nrf2 travels to the nucleus in reaction to oxidative stress and triggers a number of antioxidative enzymes, neutralizing ROS and eventually promoting homeostasis of cellular systems [44]. Nrf2 can also respond to oxidative stress by creating NADPH and increasing the expression of NADPH-producing enzymes, such as isocitrate dehydrogenase as well as glucose metabolism regulators [45].

Initiation of cancer can be prevented by Nrf2 since it minimizes oxidative stress. On the other hand, prevention and/or inhibition of Nrf2 pathway would kill cancer cells by promoting ROS. The main challenge post cancer chemotherapy is recurrence. In this regard, Nrf2 plays chemopreventive role by downregulating ROS to prevent proliferation of the remnant cells. Cancer initiation is a ROS-driven process whereby ROS trigger inflammation, promote transforming growth factor beta (TGF-β) and angiogenesis. Clearly, for chemotherapeutic activities, Nrf2 needs to be inhibited whereas to prevent cancer initiation/recurrence, Nrf2 cascade needs to be activated.

**Fig. 1:**
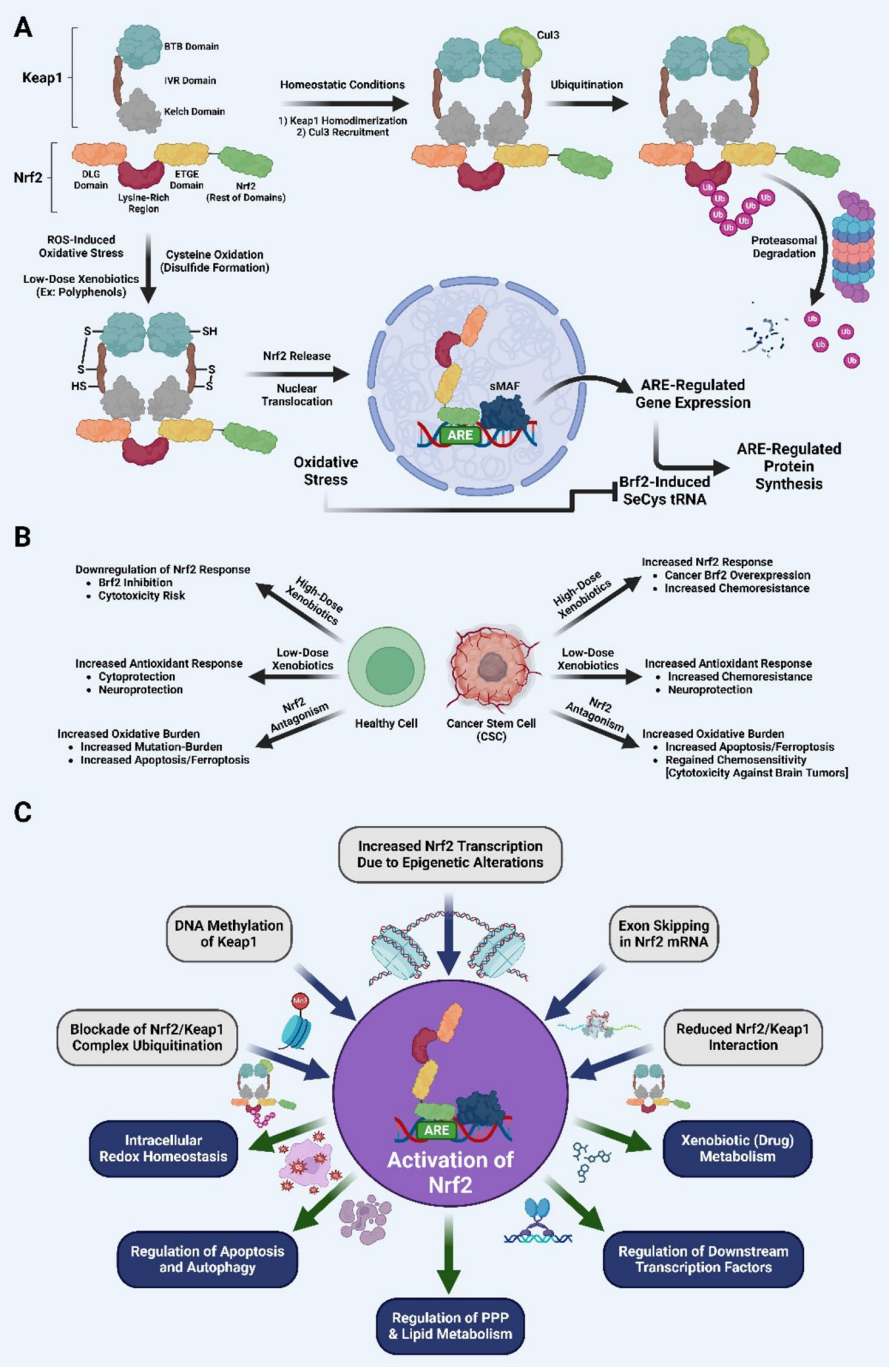
Schematic representation of the Nrf2/Keap1/ARE signaling pathway. (**A**) During normal homeostasis, Nrf2 binds to Keap1 by DLG (low affinity) and ETGE (high affinity) motifs forming a homodimer, and activates Cul3-mediated ubiquitination in the lysine-rich region followed by proteasomal degradation. Under oxidative stress or xenobiotic exposure disulfide bonds form within Keap1, modifying IVR conformation and disrupting Kelch-DLG/ETGE binding. In absence of ubiquitination, increased amount of Nrf2 protein accumulates and translocates into the nucleus. Inside the nucleus, Nrf2 forms a heterodimer with sMaf protein and subsequently binds to ARE, to trigger the transcription of downstream genes. ARE-regulated mRNA transcripts are translated with SelCys tRNA, which is directly regulated by Brf2 activity. Oxidative stress inhibits Brf2 from producing SeCys tRNA, functionally providing negative feedback on oxidative-induced antioxidant responses. (**B**) Healthy cells and cancer stem cells act differently when exposed to high and low levels of Nrf2 agonism. At low doses of xenobiotic exposure, Nrf2 stimulation increases cytoprotection/neuroprotection in healthy cells and enhances chemoresistance in cancer cells. High-dose xenobiotic exposure or ROS burden in healthy cells inhibits Brf2 activity, allowing for cytotoxicity and regulated cell death. In cancer cells high-dose xenobiotic exposure enhances chemoresistance due to cancer-specific Brf2-overexpression. Nrf2 antagonism in both health/cancerous tissue increases oxidative burden, enhancing apoptosis/ferroptosis. In cancerous cells Nrf2 antagonism helps regain chemosensitivity, inhibiting cancer-specific mechanisms which suppress chemotherapy-induced cytotoxicity. (**C**) Regulation of Nrf2-associated activities with regard to cancer. Epigenetic alterations lead to transcriptional enhancements in Nrf2 levels. Methylation of Keap1 transcriptionally reduces Keap1 levels. Exon skipping of Nrf2 and somatic mutations in either of Nrf2, Keap1, and Cul3 disrupt Nrf2/Keap1 interactions. Modifications in Keap1 cysteine-residues and Keap1‐ competing protein e.g. p62 lead to the reduced binding affinity between Nrf2‐Keap1, and antagonize ubiquitination of Nrf2. Downstream activities of Nrf2 cascade include maintenance and/or regulation of cellular redox homeostasis, apoptosis, and autophagy. Generation of NADPH, and pentose synthesis is controlled by Nrf2 downstream genes. Products of lipid metabolism serve as important biomarkers regarding cancer progression. In addition, Nrf2 promotes expression of genes involved in drug metabolism, thus playing vital role in resistance to chemotherapeutic agents. ARE, antioxidant responsive element; Brf2; B-related factor 2; IVR, intervening region; sMaf, small musculoaponeurotic fibrosarcoma protein

Nrf2 regulates the redox status of GSH homeostasis by directly controlling the catalytic and modifying subunits of glutamate-cysteine ligase involved in GSH production [46]. Also, Nrf2 regulates the transcription of many ROS-detoxifying enzymes, including Gpx2, GSTs (GSTA 1,2,3,5, GSTM 1–3, and GSTP1), as well as a GSH-based antioxidant system, thioredoxin 1, thioredoxin reductase 1, and thioredoxin-inhibitor [47–50]. Nrf2 activity enables the production of enzymes that protect against xenobiotics and oxidative stress while also catalyzing reductive processes. Nrf2 is a modular protein with seven functional domains called the Nrf2 ECH homology (Neh) domains (Neh1–Neh7) [51, 52]. The DLG and ETGE motifs present on Neh2 act as binding sites to interact with Keap1. Keap1, a Cul3-containing E3 ubiquitin ligase substrate adaptor, interacts with Nrf2 to regulate its stability. The BTB domain of Keap1 interacts with Cul3 and facilitates homodimerization of Keap1, which in turn facilitates ubiquitination and proteasomal destruction of Nrf2 [53]. The Kelch/DGR domain contains six Kelch repeatitions that interact with the ETGE and/or DLG motifs of Neh2 domain of Nrf2 [54]. Nrf2 is regulated at rest by proteasomal degradation mediated by Keap1. Nrf2 is largely found in the cytoplasm and forms a dimer with Keap1, promoting Nrf2 ubiquitination, and subsequent proteolysis (Fig. 1A).

Clearly, under normal conditions, Keap1 strictly regulates Nrf2 expression to a low level in order to avoid overexpression of its target genes [55]. Upon exposure to oxidative stress, Nrf2 translocates to the nucleus, forming a heterodimer with small Maf, and binds to ARE sequences to regulate the transcription of several genes (Fig. 1A). In the downstream cascade, these genes are involved in intracellular redox homeostasis, apoptotic signaling, metabolism, and autophagy. Nrf2-mediated antioxidant response is amongst the major cellular defense mechanisms favoring cell survival under stress-insults. However, response to Nrf2 modulation is not exactly similar in normal cells and tumorigenic cells (Fig. 1B).

Emerging evidence suggests that constitutive activation of Nrf2 (Fig. 1C) promotes the expression of genes involved in drug metabolism, thereby imparting resistance to foreign chemotherapeutic agents and allowing cell survival [56]. Also, Nrf2 supports cell proliferation by inducing metabolic reprogramming towards anabolic pathways, augmenting purine synthesis, and influencing the pentose phosphate pathway (PPP). Nrf2 redirects glucose and glutamine into anabolic pathways under the influence of PI3K-Akt signaling [57]. Interestingly, gain of Nrf2 signaling can promote tumorigenesis via an autoregulatory feedback loop utilizing miRNA-dependent regulation of PPP and HDAC4. Furthermore, through upregulation of Bcl-2 and Bcl-xL, two ARE-controlled genes in mammalian cells, Nrf2 can exert somewhat protection from apoptosis to cells, which potentially increases its oncogenic potential. All these Nrf2-mediated pathways are likely to contribute to the maintenance of redox balance, and promotion of tumor growth. This indicates that targeting them may improve the therapeutic outcome in cancer patients. On the other hand, Keap1 negatively regulates the activities of Nrf2. Under basal conditions, Keap1 constantly subjects Nrf2 to ubiquitin-dependent degradation to maintain low intracellular Nrf2 levels (Fig. 1A). Keap1, can sense an imbalance in redox homeostasis to modulate the Nrf2-mediated response. On exposure to oxidative stress, modification of three major Keap1 cysteine residues, Cys151, Cys273, and Cys288 (that are responsible for the ‘sensor’ functionality) imposes a conformational change to disrupt the weak binding between Kelch domain of Keap1 and DLG motif of Nrf2, resulting in reduced ubiquitination of Nrf2 without complete dissociation of Nrf2 from Keap1 [58]. Consequently, high amount of Nrf2 protein cumulates and translocates into the nucleus. Nrf2 plays the role of the pathway’s executor by activating gene expression through the process of binding to ARE. Consequently, available Nrf2 level increases leading to subsequent Nrf2-mediated cell signaling events. Notably, only genes that include an ARE in their promoter region are implicated in the Nrf2/Keap1/ARE signaling cascade [59].

**Fig. 2:**
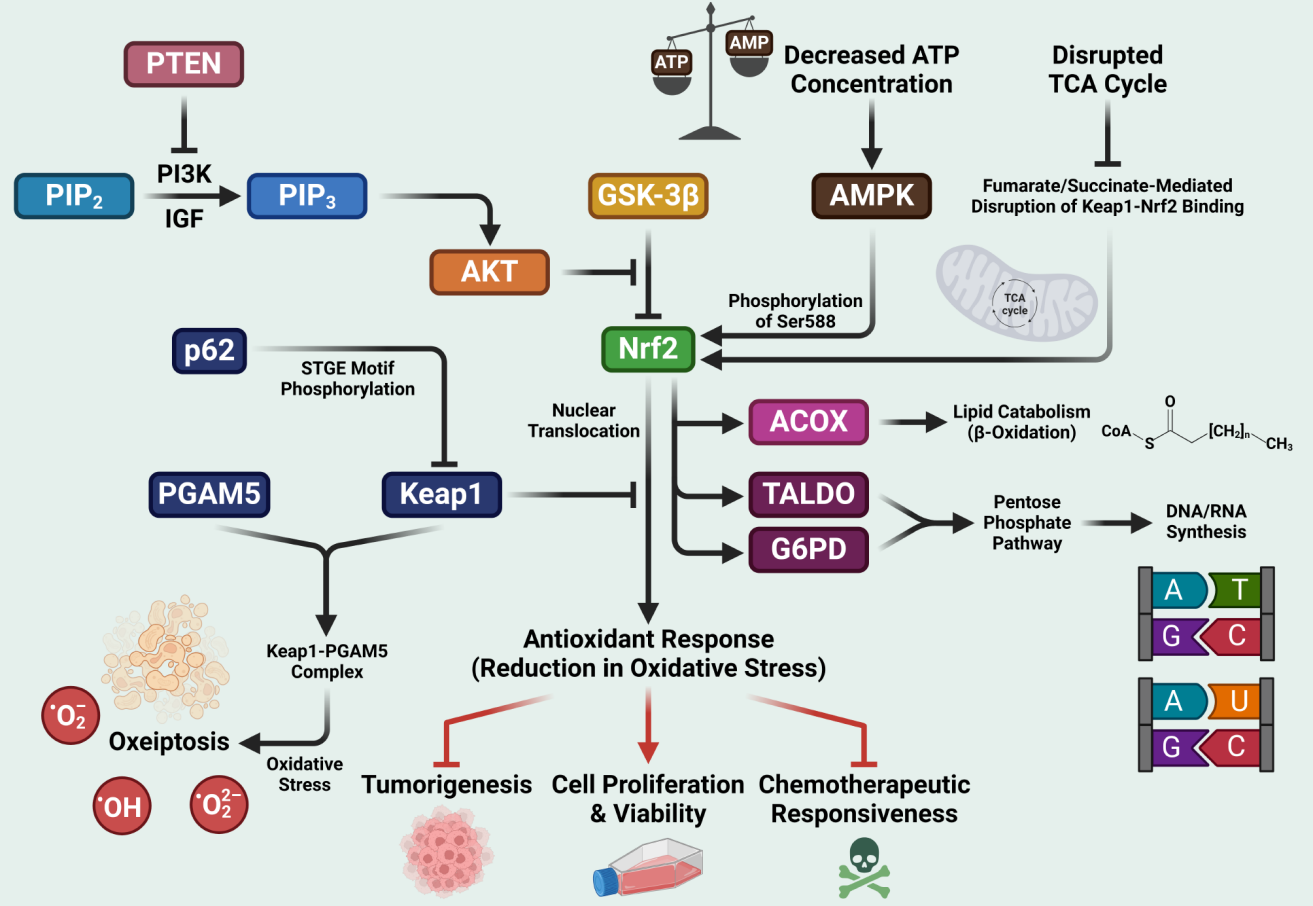
Schematic representation of pathways influencing Nrf2 build-up and its downstream pathways. PI3K-Akt pathway prevents non-Keap1 degradation of Nrf2, p62/SQSTM pathway inhibits Keap1 to facilitate nuclear translocation of Nrf2, AMPK pathway leads to Nrf2 build-up by Nrf2 serine 588 phosphorylation, disrupted TCA cycle leads to diminished levels of Nrf2. Nrf2 increases glucose uptake and facilitates PPP, breaks down lipid, and diminishes tumorigenesis in cells. However, Nrf2 leads to increased cell proliferation and decreased chemotherapeutic responsiveness in cancer cells. Keap1 forms complex with PGAM5 and under oxidative stress leads to cell death. Arrows indicate downstream processes, flat-headed line indicates inhibition. TCA, tricarboxylic acid; PGAM5, phosphoglycerate mutase family member 5; PPP, pentose phosphate pathway

Regardless of oxygen availability, cancer cells increase glucose absorption and transform glucose into lactate. Accelerating glycolytic flow allows for the production of ATP while also meeting the metabolic demands of growing cells. Cancer cells’ metabolic characteristics boost the synthesis of DNA and lipids. Despite the tumors’ abnormal activation, it appears to include a general induction against multiple pathways that support critical metabolic functions and redox balance. Nrf2 has been identified as a transcription factor to regulate antioxidant gene expression. It has also been implicated that the Nrf2/Keap1/ARE signaling cascade is associated with metabolic reprogramming in cancer cells via modulating multiple mechanisms [60, 61]. Figure 2 schematically represents upstream and downstream events involving Nrf2. Table 3 represents the apparently opposing dual roles of the Nrf2 signaling cascades regarding cancer.

**Table 3:** Dual role of Nrf2/Keap1/ARE cascade in cancer [60]

Tumor suppression	Tumor promotion
Activates cellular defense mechanism against xenobiotics and/or oxidative stress	Nrf2 activation, protects cancer cells from deleterious effects of oxidative stress and chemotherapeutic agents
Nrf2 initiates cellular defense mechanisms against stress	Activated Nrf2 is characteristic of existing tumor sites
Inactivation of Nrf2 corresponds to elevated carcinogenesis and metastasis	Nrf2 promotes survival, growth, and proliferation of cells

### PI3K/Akt-Nrf2 interactions

When growth factors are activated in normal cells, PI3K signaling, and downstream Akt and mTOR induce enhanced glycolytic flux and fatty acid synthesis [62]. The PI3K/Akt pathway also serves as a primary proliferative signal in the oncogenic pathway by interacting with the Nrf2 cascade. PI3K catalyzes the phosphorylation of PIP2 to form PIP3 when the insulin-like growth factor (IGF) receptor is active. PIP3 synthesis is required for Akt activation, which promotes downstream signaling events such as GSK-3β inhibition [63]. GSK-3β is a crucial mediator that is blocked by Akt-mediated phosphorylation, and Nrf2 is phosphorylated by GSK-3β, allowing it to be recognized by β-TrCP, which then labels Nrf2 for ubiquitination regardless of whether it is mediated by engaging the Keap1/Cul3 complex or not [4]. When GSK-3β is phosphorylated, Nrf2 cumulates due to suppression of Keap1-independent degradation and increased abundance in Nrf2, which stimulates the activation of metabolic genes as well as anabolic metabolism under the influence of PI3K-Akt signaling [50]. Evidence suggests that the PI3K pathway contributes to Nrf2 activation in a variety of circumstances [64]. When PTEN levels are low, Akt is activated while GSK-3β is suppressed. GSK-3β inhibition has been shown to downregulate phosphorylation of Nrf2, allowing Nrf2 to evade Keap1-independent and β-TrCP-Cul1-dependent destruction in the nucleus. Thus, loss of PTEN has been shown to boost nuclear accumulation of Nrf2 and Nrf2 target gene expression [65]. In line with the findings, deletion of Keap1 and PTEN leads to higher levels of Nrf2 levels within cancer cells [50]. Taken together, stimulation of the PI3K/Akt signaling pathway improves nuclear translocation of Nrf2, thus allowing Nrf2 to produce enhanced antioxidant effects that can protect tumorigenesis. On the contrary, in cancer cells, PI3K/Akt signaling cascade enhances cell proliferation, and decreases chemotherapeutic responsiveness.

### Influence of AMPK on Nrf2

In normal cells, AMPK is the master regulator of metabolism and energy homeostasis. AMPK activation has been linked to increased glucose absorption, fatty acid uptake, activation of autophagy, and enhanced ATP levels [66]. Several studies reported AMPK to enhance Nrf2 activity and phosphorylate Nrf2 serine 588 [67, 68]. AMPK activity affects the phosphorylation and inhibition of GSK-3β, which promotes Nrf2 degradation. As a result, AMPK regulates nuclear localization and stability of Nrf2 [69].

### Mitochondrial crosstalk with Nrf2/Keap1 cascade

Mitochondria are key organelles that are in charge of ATP generation and other cellular functions. Furthermore, it regulates the TCA cycle, Ca^2+^ and ROS homeostasis, fatty acid and amino acid metabolism. Mitochondrial function is influenced by Nrf2-dependent interactions in metabolism and homeostasis. Nrf2 has been demonstrated to be activated by abnormal build-up of the TCA cycle intermediates [70]. In the absence of Nrf2, glucose oxidation and substrate flow into the TCA cycle are reduced [71]. In contrast, constitutive Nrf2 activation promotes glucose oxidation and substrate flow into the TCA cycle. Silencing Nrf2 reduced ATP generation and oxygen consumption in human brain cancer cells [72]. Inactivation of fumarate hydratase in particular leads to build-up of fumarate and succinate, which disrupts the Keap1-Nrf2 binding. When Nrf2 is activated, fumarate binds with cysteine residues in the Keap1 protein [73]. Furthermore, when exposed to high levels of ROS, the PGAM5-Keap1 complex promotes oxeiptosis, a caspase-independent cell death pathway [74].

### Influence of Nrf2 on pentose phosphate pathway

The activation of Nrf2 alters purine biosynthesis through pentose phosphate pathway (PPP) [75]. Nrf2 favorably regulates the production of transaldolase 1 (TALDO1) and transketolase (TKT) in non-oxidative branch of PPP, and is also involved in direct transcriptional regulation of PPP-related enzymes such as Glucose-6-phosphate dehydrogenase (G6PD) and phosphogluconate dehydrogenase (PGD) that catalyze the processes in the oxidative phase of PPP [76, 77]. G6PD, PGD, TKT, and TALDO1 promote glucose flux and create purines, which are building blocks for DNA and RNA, and has also been implicated to aid in cancer proliferation [50]. Nrf2 also promotes purine base synthesis through the indirect regulation of phosphoribosyl-pyrophosphate amidotransferase (catalyzes the rate-limiting step in purine biosynthesis), and methylenetetrahydrofolate dehydrogenase 2 (a nuclear-encoded mitochondrial enzyme with methylenetetrahydrofolate dehydrogenase and methenyltetrahydrofolate cyclohydrolase activities) [50].

### p62/SQSTM-mediated regulation of Nrf2/Keap1 cascade

Emerging evidence indicates that p62/Sequestosome 1 protein (p62/SQSTM) can regulate Nrf2 activity [60, 78, 79]. p62/SQSTM1 is a scaffold protein that governs the autophagy pathway by selecting cytoplasmic aggregators of ubiquitinated proteins and organelles. p62 includes a STGE motif resembling the Nrf2 ETGE motif. Thus, it interacts directly with the Kelch domain of Keap1, and p62 accumulation causes serine phosphorylation of a STGE motif [80]. This phosphorylation boosts p62/Keap1 association, aggregation, and autophagic destruction, resulting in stabilization of Nrf2. However, during defective autophagy, this adaptive response can become pathogenic, resulting in the formation of cytoplasmic protein inclusions in a p62- and Nrf2-dependent manner [81]. This is significant since p62 gene amplification and abnormal build-up of phosphorylated p62 protein have been linked to the acceleration of cancer growth [50].

### Regulation of lipid metabolism by Nrf2

Nrf2 regulates the oxidation of fatty acids by controlling the expression of two peroxisomal enzymes, acyl-CoA oxidase 1 and 2 (ACOX1 and ACOX2, respectively) [50]. Nrf2, on the other hand, regulates activities such as lipid production, fatty acid desaturation, and fatty acid transport [82]. Findings imply that Nrf2 may negatively influence lipid production and decrease NADPH consumption in cancer cells. Nrf2 transcriptionally regulates fatty acid oxidation-related genes and stimulates lipid breakdown, lowering the formation of NADPH in cancer cells [50]. These findings suggest that the Nrf2/Keap1/ARE signaling cascade can modulate lipid metabolism, including β-oxidation of fatty acids, thus playing a role in the regulation of oxidative stress.

### Nrf2 in glioblastoma stem cell-mediated malignancy

Research has continued to highlight Nrf2 as a key factor in brain-cancer development and progression owing to its influence on cancer stem cells. Glioblastoma exhibits a functional cellular hierarchy supported by self-renewing glioblastoma stem cells (GSCs) [83]. The fact that Nrf2 is overexpressed in CD133 + glioma stem cells compared to CD133 − stem cells suggests that Nrf2 expression may contribute to the malignant proliferation and differentiation of GSCs [84]. Evidence from patient-derived GSC suggests that TGF-β upregulates the production of NOX4 protein, which is also observed in other cancer types such as hepatocellular carcinoma [85]. GSCs have also been linked to poor prognosis and chemoresistance [86–88]. The correlation between promotion of cell proliferation and onset of malignancy with Nrf2 expression suggests enhanced purine synthesis and antioxidant protection of DNA may enhance rate of proliferation and eventual malignancy from GSCs.

### miRNA in Nrf2 regulation

miRNAs are small non-coding RNA strands that regulate gene expression by attaching to particular mRNA molecules [89]. Exon-skipping of Nrf2 disrupts Nrf2/Keap1 interactions. The first miRNA implicated in Nrf2 modulation was miR-144, whose expression suppressed Nrf2 [90]. **C**ancers have exhibited miRNA-mediated downregulation of Nrf2, which may be related to the underlying disease mechanism [91, 92]. Experimental findings suggest that upregulated miRNAs such as miR-323/miR-326/miR-329, and miR-130a/miR-155/miR-210 could be related to downregulation of Nrf2 and subsequent increments in cell death leading to reduced mortality in glioblastoma patients [93]. Shifting levels of miRNA-128 and miRNA-342 may impart ramifications for the histopathological grading of glioblastoma [94]. Interestingly, a number of miRNAs display altered levels in several glial cell types, further demonstrating the significance of miRNA in the progression of glioblastoma [95]. Upregulation of miR-221, miR-204, and miR-16, and downregulation of miR-195, miR-633, and miR-136 are implicated in glioblastoma [95].

## Role of Nrf2 in phenotype switch and treatment resistance

The heterogeneity of glioblastoma tumors is thought to contribute to their very aggressive nature. Through clinical experience and extensive molecular investigation of tumors, clinically significant subgroups of glioblastoma with similar genetic expression and methylation profiles have been identified [96, 97]. Glioblastoma tumors are widely classified into four subtypes: proneural, neural, classical, and mesenchymal. Later on, glioblastoma tumors have been discovered to be heterogeneous in terms of cell subtypes, with many populations of cells resistant to many drugs and radiotherapy [98]. Multidrug-resistant phenotypes have been related with the mesenchymal profile [99]. This subtype of glioblastoma cells is extremely invasive and usually displays neural stem cell markers [97, 100]. The gene enrichment study of these tumors indicated overexpression of pathways related with mesenchymal transition, extracellular matrix receptor responses, antigen processing and presentation, ATP-binding cassette transporters, and drug metabolism [99]. These characteristics of cancer cells are essential to their endurance and resilience. It is interesting to note that certain treatment modalities, like radiation and chemotherapy, may result in a shift to a mesenchymal phenotype [100].

According to gene expression analysis of glioblastoma samples using microarray and RNA-sequencing, patients with tumors exhibiting elevated levels of Nrf2 correspond to lower survival times [101]. Furthermore, it has been discovered that Nrf2 activity and tumor grade are interlinked, with no patient with grade I tumors exhibiting elevated Nrf2 levels [101]. The scientists have also identified a potential mechanism for promoting mesenchymal phenotype by means of co-regulatory positive feedback between Nrf2 and the autophagy regulating protein complex SQSTM1/p62. It has been proposed that by interacting with their enhancer DNA area, Nrf2 could directly stimulate the expression of Slug and ß-catenin mesenchymal markers [101]. The findings have been supported by linking the overexpression of p62 in the mesenchymal transition to the TLR4–p38–Nrf2 pathway [102]. Additionally, Nrf2 increases resistance of glioblastoma to redox anticancer agents. By activating the Nrf2-mediated antioxidant response, glioblastoma cells could develop resistance to cannabidiol [103]. Resistant cells expresses mesenchymal markers such as TNSFR10, CEBPB, and CD44, thus an adaptive reprogramming toward a mesenchymal phenotype and an Nrf2-mediated antioxidant response could be the causes of the resistance.

## Plant secondary metabolites: new prospect in brain cancer therapy

An imbalance between the generation and accumulation of ROS and RNS leads to oxidative stress. Numerous studies found a link between oxidative stress and the emergence of brain tumors [11, 104]. Plant secondary metabolites represent a great reservoir of potential chemotherapeutic agents against cancers including brain cancer. Despite advances in operative and postoperative procedures, it is almost impossible to completely resect the tumors due to their invasive and diffuse nature. Several natural, plant-derived products, however, have demonstrated promising preventive and/or therapeutic effects, such that they might serve as potential resources for anticancer drug discovery. Emerging evidence reveals that regulating Nrf2/ARE and downstream antioxidant enzymes by the use of Keap1 modulators, might play pivot in the management of brain tumors [105–107]. Many naturally occurring plant secondary metabolites have been recognized for chemotherapeutic and chemopreventive potential against cancers [108]. Natural product-derived inhibitors of the Nrf2 pathway cause an increase in ROS production in cancer cells, which may lead to cell death in ROS-sensitive cancer cells. Importantly, through the downregulation of detoxification enzymes and drug excretion transporters, they frequently make malignancies more sensitive towards chemotherapies and radiation [109]. On the other hand, some nature-derived phytochemicals endorse Nrf2 signaling to prevent cancer initiation and/or recurrence [110]. The influence of numerous plant secondary metabolites on cancer initiation, progression, and metastasis has been assessed in a number of studies. Furthermore, research has revealed that using nature-derived substances in conjunction with established radiotherapy and chemotherapy can exert synergistic benefits, reducing side effects and improving treatment outcomes [111]. Some of them can also cross the BBB, a feature that is crucial in the design of therapies for CNS tumors [112].

## Scopes of nature-derived compounds in modulating Nrf2/Keap1/ARE axis

It has already been discussed that for chemotherapeutic activities, Nrf2 needs to be inhibited while to prevent cancer initiation and recurrence, Nrf2 cascade needs to be activated. Activation of Nrf2 cascade minimizes oxidative stress to prevent cancer initiation. On the contrary, downregulation of Nrf2 signaling cascade increases oxidative stress, which can kill ROS-sensitive tumor cells. Again, hyperactivation of Nrf2 cascade endorses survival of both normal and malignant cells by protecting from oxidative stress [60]. Nrf2/Keap1/ARE pathway plays a major role in cancer prevention by anti-inflammatory effects, and reducing oxidative stress to reduce DNA damage [113]. Apart from proliferative effects, activation of Nrf2 also leads to increased antioxidative enzymes through Nrf2/Keap1/ARE pathway which counteracts ROS mediated cell death [114]. Table 4 represents various downstream activities mediated by Nrf2 signaling cascade. Multiple plant-derived secondary metabolites are involved in the modulation of Nrf2 signaling cascade through controlling the interaction between Nrf2 and Keap1 [6, 115]. These nature-derived molecules are believed to provide a rather safe method of anticancer interventions compared to their synthetic counterparts, leading to a new horizon of chemoprevention and therapeutics. Some of the compounds even exhibit a relatively high level of penetration through BBB [116–118]. BBB forms a selectively permeable barrier that restrict entry of many therapeutic molecules into the CNS. Majority of anticancer agents are unlikely to pass beyond BBB owing to hydrophobicity, large molecular weights and enzymes present in the BBB [11]. Therefore, ability of some Nrf2-modulators to cross BBB becomes crucial in brain cancer therapeutics. Hence, plant secondary metabolites represent a rather less explored pool of possible therapeutic and/or preventive modalities against different types of cancers, including brain cancer. Thus, they seem to be potent competitors that might fill in the void created by concerns mentioed in Table 1.

**Table 4:** Downstream functions mediated by Nrf2- associated signaling cascade

Activities	Biomolecules involved	References
Apoptosis and autophagy	Bcl-2, Bcl-xl, p62	[81]
Catalytic degradation of xenobiotics	Aldo-keto reductase, carbonyl reductase, NQO1, alcohol dehydrogenase, aldehyde dehydrogenase, cytochromes, SOD, epoxide hydrolases, carboxyl esterases	[75]
Efflux of xenobiotics	Multidrug resistance associated proteins, P-gp	[75]
GSH metabolism	Glutamate-cysteine ligase, GPX, glutathione reductase, xCT	[46]
Heme metabolism	Heme oxygenase 1, biliverdin reductase, ferrochelatase	[75]
Metabolism of fatty acids	ATP-citrate lyase, acetyl-CoA carboxylase, fatty acid synthase, stearoyl CoA desaturase	[50, 62, 82]
Purine biosynthesis	Phosphoribosyl pyrophosphate amidotransferase, methylenetetrahydrofolate dehydrogenase 2	[57]
Redox homeostasis	Glutamate-cysteine ligase, Gpx,, thioredoxin 1, thioredoxin reductase, peroxyredoxin 1, sulfiredoxin 1	[3, 60]
Regulation of PPP	G6PD, 6-phosphogluconate dehydrogenase, isocitrate dehydrogenase 1, transketolase, transaldolase 1, aldolase 1	[57]
Regulation of transcription factors	Aromatic hydrocarbon receptor, PPARγ, RXRα, CEBPα	[60]
Thioredoxin-mediated reduction reactions	Peroxiredoxins, thioredoxin 1, thioredoxin reductases	[47–50]
Xenobiotic detoxification by conjugation	UGT, GST, sulfotransferases, N-acetyl transferase	[60, 75]

### Chemopreventive role of Nrf2 activators

Under normal conditions, Nrf2 maintains cellular redox equilibrium and exerts anti-inflammatory and anticancer effects, hence promoting cell survival [75]. Inflammation leads to the production of ROS and RNS, resulting in DNA damage, activation of oncogenes and inactivation of tumor suppressor genes, initiation of cell proliferation, metastasis, and angiogenesis. Cytokines with pro-inflammatory characteristics are implicated in neuroinflammation-induced carcinogenesis [75]. NF-κB mediates transcription of pro-inflammatory cytokines and enzymes such as cyclooxygenase-2 and inducible nitric oxide synthetase (iNOS). These enzymes play a role in the promotion of both carcinogenesis and angiogenesis [119]. Nrf2 inhibits nuclear translocation of NFκB and lowers ROS [120]. Hence, Nrf2 imparts chemopreventive effect through prevention of initiation of inflammation and arresting the transcription of inflammatory cytokines [121]. Furthermore, overexpression of HO-1, a downstream target of Nrf2, reduces TNF-α-induced oxidative stress and IL-3 via suppressing DNA-binding ability of activator protein-1 [120].

One of the most essential mechanisms in antitumorigenesis is the activation of Nrf2. Nrf2 is activated in the tumor microenvironment by tumor suppressor genes BRCA1 and protein p21 via suppression of Keap1/Nrf2 complex formation, and is inhibited by oncogene Fyn-mediated degradation [122]. Many studies demonstrated that Nrf2 deficiency is positively correlated with carcinogenesis [75, 123, 124]. Nrf2 activator luteolin has been used as a preventive measure against glioma in studies [125].

Under normal physiological conditions, activation of Nrf2 in non-malignant cells has been demonstrated to exert cancer chemopreventive effects [126]. This is primarily accomplished by regulating redox homeostasis, which ensures genomic stability and cell survival via the actions of antioxidant defense enzymes e.g. SOD, CAT, GPx, GSH synthase, GST, thioredoxin, and glutathione reductase, and phase 2 and 3 detoxifying enzymes [127]. These expressed proteins prevent oxidative stress-induced DNA damage by either decreasing the exposure of DNA to carcinogens (both exogenous and endogenous) or increasing the rate at which carcinogens are detoxified [126]. Inhibiting the activation of pro-carcinogens is another way to protect DNA. Due to this, the inactivation of the Nrf2 pathway can lead to an increase in oxidative stress, as well as mutagenesis, carcinogenesis, and the formation of tumors in normal cells [128]. Viewing from the results of numerous studies involving the use of plant secondary metabolic products as an activator of the Nrf2-mediated signaling pathway, we can consider this approach as a potential strategy in prevention of brain cancer.

A wide variety of phytochemicals, including flavonoids, sulforaphanes, alkaloids, and polyphenols, have been found to activate Nrf2 [129]. Disrupting Keap1 and Nrf2 connections, phosphorylating Nrf2, and blocking Nrf2 ubiquitination are the key mechanisms responsible for activating the Nrf2 cascade [130, 131]. The transcription of ARE-associated cytoprotective genes is facilitated by nuclear translocation of Nrf2, and several of the activators aid in this process [132]. Electrophilic canonical activators that interfere with the Keap1/Nrf2 association oxidize or alkylate the cysteine residues of Keap1 to release Nrf2 from the protein [64]. Clinical trials have revealed that resveratrol can disrupt Nrf2-Keap1 relationships by conformational changes; for instance, 500 mg/day dose of resveratrol for 30 days is effective in reducing chronic subclinical inflammation and improving redox state by electrophilic mutations to the Keap1-Cys151 thiol group [131]. The stabilization of Keap1 and Nrf2 is disrupted as a result of post-translational changes in Cys151, which in turn prevents the ubiquitination and proteasomal degradation of Nrf2 by facilitation of ARE mediated gene transcription [65]. By inhibiting Nrf2 from binding to Keap1, SQSTM activates the Nrf2 pathway in a non-canonical fashion [133]. SQSTM increases the autophagic degradation of Keap1 and also competes with Nrf2 for binding to Keap1, preventing the formation of the Nrf2-Keap1 complex [133, 134]. Silencing p62 results in a substantial upregulation of Keap1 and a downregulation of Nrf2 at the mRNA and protein levels, respectively, indicating that p62 may be effective in downregulating Keap1 protein via autophageal breakdown [133]. The majority of endogenous activators of the Nrf2/ARE pathway dissociate Nrf2 from Keap1 by increasing Nrf2 phosphorylation [135]. Dissociation of Nrf2 from Keap1 has been observed after PERK-mediated direct phosphorylation of Nrf2 in experimental models [76]. Similarly, phosphorylation of Nrf2 occurs on serine (Ser212, Ser400, Ser558, Ser577) and threonine (Thre559) residues in HEK 293T cells by activating Janus kinases 1 and 2 (JNK1/2), extracellular ERK2, and p38 [126]. It has been hypothesized that phytochemicals like diallyl sulfide can stimulate the aforementioned endogenous activators of the Nrf2/ARE pathway. Diallyl sulfide promotes the dissociation of Nrf2 from Keap1 and its subsequent nuclear translocation via phosphorylation of ERK and p38 [136]. Nuclear translocation of phosphorylated Nrf2 appears to be facilitated by its endogenous activators, the protein kinases [137]. Akt, a downstream regulator of PI3K, is implicated in the activation of the Nrf2/ARE pathway [138]. Akt promotes nuclear translocation of Nrf2 and subsequent transactivation of ARE genes [139]. Protein disulfide isomerase (PDI) is an overexpressed protein in glioblastoma that contributes to rapid progression of the disease. In a study, PDI inhibitor pyrimidotriazinedione 35G8 suppressed PDI target genes such as EGR1 and TXNIP in human glioblastoma cells, activated Nrf2-mediated antioxidant response and ER stress response [140]. These, in turn, led to glioma cell death by a combination of ferroptosis and autophagy [140, 141]. Wogonin displays antioxidant and anti-inflammatory actions owing to its ability to trigger Nrf2 expression [142].

Concerns regarding side effects of Nrf2 activators calls for further explorations. Omaveloxolone, an Nrf2 activator, confers few off-site mild side effects such as upper respiratory tract infections, nasopharyngitis, fatigue, diarrhea, and nausea [143]. Another Nrf2 activator dimethylfumarate leads to decrease in number of lymphocytes [144]. Some electrophilic Nrf2 activators imply off-target effects, which can be explained by non-specific S-alkylation of cysteine thiols and subsequent depletion of anti-oxidative glutathione [145]. Majority of the side effects does not result in serious or long term consequence since they can be managed by selecting an alternative or careful formulation-design and/or dosage regimen. However, the main concern regarding side effects of Nrf2 activation lies in its desired mechanism itself. As Nrf2 activation promotes antioxidant defense, it hinders the possibility of cancer cell destruction through oxidative stress-mediated apoptosis, thus leading to chemoresistance and radioresistance in cancer cells [75]. This calls for more attention than other side effects and research oriented to optimize the dosage of Nrf2 activators and accurate site specific delivery might be able to do away with such complication.

### Chemotherapeutic role of Nrf2 inhibitors

Nrf2/ARE pathway promotes development and metastasis of cancer through diverse mechanisms. Nrf2/Keap1/ARE pathway aids in cancer cell growth and proliferation by promoting detoxification and antioxidant defense. The pathway is also involved in regulating cell cycle, hence, enhances activities of proliferation-associated genes such as Bmpr1a, Igf1, Itgb2, Jag1. Cell cycle arrest in G2/M phase is observed with deficiency of Nrf2, i.e. blockade of Nrf2/ARE pathway would supposedly lead to antiproliferative effect on cancer cells [146]. Apart from proliferative effects, Nrf2 activation enhances antioxidative enzymes, which counteracts ROS-mediated cell death. Thus, underfunctioning of Nrf2 would lead to cell death in ROS-sensitive cancer cells. One of the hallmarks of inflammation-induced carcinogenesis is the activation of angiogenesis. Overexpression of HO-1 increases VEGF production and VEGF-mediated angiogenic activity by enhancing proliferation, migration, tube formation, and capillary outgrowth from endothelial spheroids. In glioma, hypoxia-induced activation of HIF-1/VEGF signaling has been blocked experimentally by Nrf2 inhibition, resulting in suppression of angiogenesis [147]. Nrf2/Keap1/ARE pathway activation also leads to enhanced chemoresistance and radioresistance in cancer cells [75]. Thus, inactivation/blockade of the Nrf2 cascade seems promising towards cancer therapy.

Pretreatment with melatonin has been observed to reverse methamphetamine-stimulated NF-κB response via increased Nrf2 activation causing an increase in cell viability and proliferation in glioma cells [148]. Numerous small molecules isolated from natural sources possess inhibitory effect on Nrf2 cascade. Wogonin reverses chemoresistance to exert anticancer effects by decreasing Nrf2 activity through downregulation of PI3K/Akt and Stat3/NF-κB signaling [149, 150]. Although wogonin has minimal toxicity and good pharmacokinetic features, it plays opposing roles in Nrf2 modulation [142]. The dual role of wogonin on Nrf2 cascade clearly calls for further studies to arrive at a decisive conclusion regarding optimization and probableble clinical translation [142, 149]. It has been discovered that certain medications now in use to treat a wide range of disorders inhibit Nrf2 function. All-trans-retinoic acid (ATRA) has been proposed as a specific Nrf2 inhibitor, as it prevents Nrf2 from forming a complex with retinoid X receptor α, thereby blocking activation of the Nrf2 pathway and suppressing chemoresistance [151]. The bioactive component of the traditional Chinese medicine herb *Dichroa febrifuga* is called febrifugine, from which halofuginone is derived. Phase II clinical studies for cancer and fibrotic illnesses have been completed of this drug. Although halofuginone is not a particular inhibitor of Nrf2, it has recently been shown that it can reduce Nrf2 protein synthesis via inhibiting prolyl-tRNA synthetase [61]. Rutaecarpine, a plant secondary metabolite has also exhibited therapeutic promise against neuro-oxidative damage by modulating the Nrf2/Keap1 axis [152]. However, halofuginone co-treatment illustrates a unique therapeutic strategy to surpass Nrf2-mediated chemoresistance in a xenograft tumor model [153].

Emerging evidence suggests that inhibition of the Nrf2/Keap1/ARE pathway provides chemotherapeutic effects against cancers including brain cancer [146, 154, 155]. Risks associated with Nrf2 inhibition seems an important factor as Nrf2 inhibition largely compromises antioxidant defense in cells. This, almost single-handedly hinders the Nrf2 inhibitors to be translated from laboratories to clinics. Nrf2 inhibition would lead to non-specific oxidative stress, which may lead to a number of complications in human body including cardiovascular complications, wound healing impairment, risk of inflammation, risk of diabetes, possibility of carcinogenesis in normal tissues due to extrinsic ROS build-up and enhanced oxidative DNA damage, and in context of brain it aids neurodegeneration and gives rise to several neurodegenerative diseases as well [156]. Hence, to destroy “Nrf2 addicted” cancer cells in advanced stage, a careful approach is required with target specific delivery to do away with such therapeutic constraint.

## Modulation of Nrf2 signaling cascade by plant secondary metabolites

**Fig. 3:**
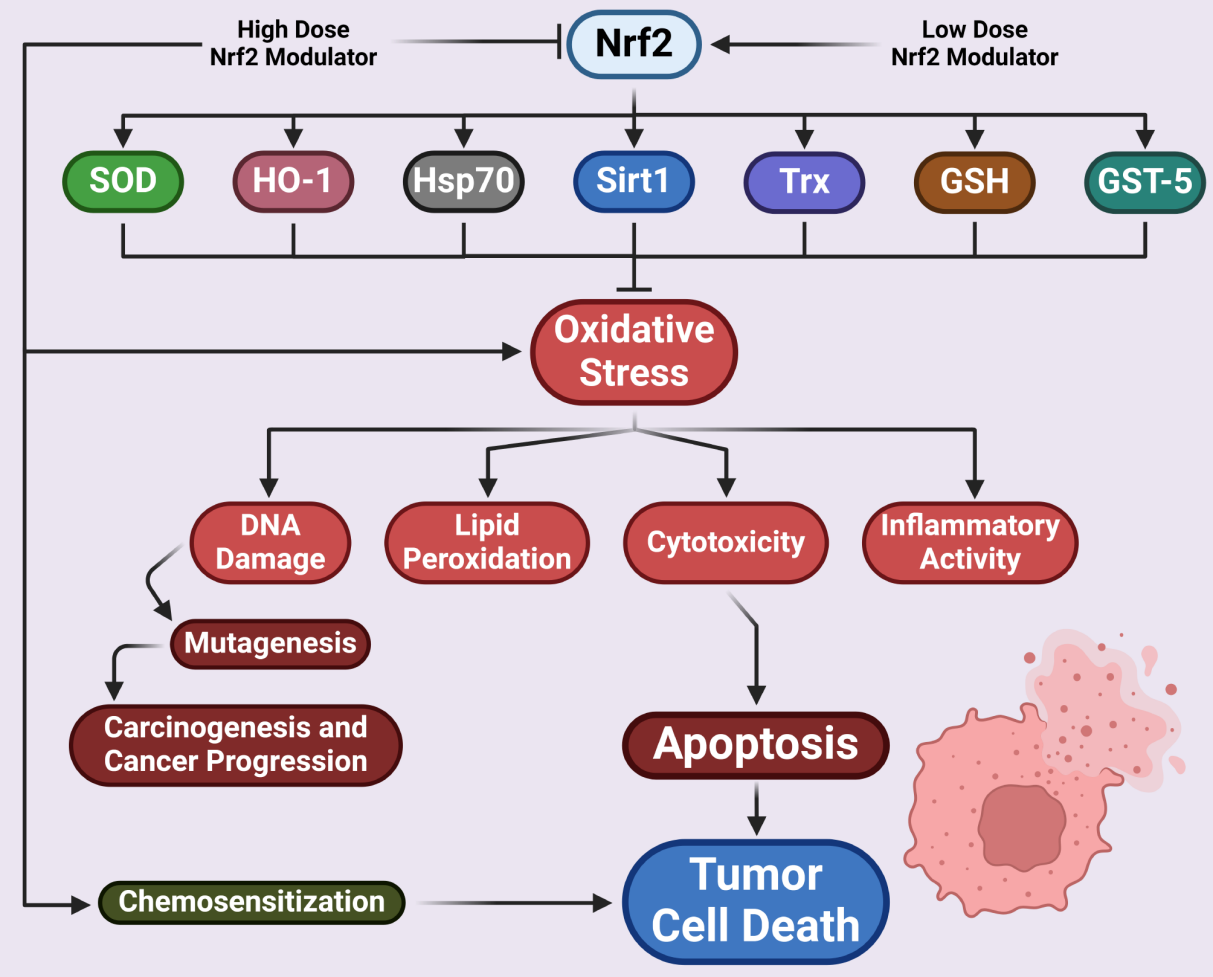
Dose-dependent regulation of the Nrf2 cascade by phytochemicals. Nrf2 modulators at low doses activates Nrf2 which further enhances activation of vitagenes such as SOD, HO1, Hsp70. Sirt1, Trx, GSH and GST4 which reduces oxidative stress leading to reduction in lipid peroxidation, DNA damage, inflammatory activation and induction of cytotoxicity. High dose of polyphenols however, leads to depletion in Nrf2 levels causing increase in oxidative stress leading to higher cytotoxicity in tumor cells; they compromise Nrf2 mediated cytoprotection of tumor cells and enhances chemosensitivity leading to tumor cell death. Arrows indicate downstream processes, flat-headed line indicates inhibition. GSH, reduced glutathione; Sirt1, sirturin 1; SOD, superoxide dismutase; Trx, thioredoxin

Numerous studies indicate that different nutraceuticals possess powerful antioxidant and anti-inflammatory properties for maintaining cellular redox homeostasis by activating antioxidant defense pathways such as Nrf2 and its phase II detoxification genes, also known as vitagenes. Vitagenes such as HO-1, Hsp70, Sirt1, Trx, SOD, GSH, and GST4 regulate signaling cascades during oxidative stress and pro-inflammatory cytokines in a variety of disorders, including brain tumors [42, 157–160]. It is worth noting that therapeutic compounds, especially polyphenols, found in plants adhere to the concept of hormesis, which is a biphasic dose-response process whereby small to moderate stress are utilized to trigger cellular adaptive reactions that protect biological systems from subsequent massive and possibly lethal stresses [161–164]. As a result, dietary polyphenols can be classified as hormetic nutrients since they can induce contrasting effects in a dose dependent manner (Fig. 3). Low doses of such compounds activate antioxidant pathways for neuroprotection, preventing or attenuating oxidative damage and inflammatory responses whereas high doses lead to the opposite effect. Emerging research suggests that low-dose hormetic nutrients upregulate the antioxidant Nrf2 pathway and vitagenes, enhancing stress resilience and preventing or even treating oxidative stress-related brain diseases [165, 166]. On the other hand, large doses of nature-derived compounds can be harmful, inhibiting antioxidant pathways and activating indicators of oxidative stress and lipid peroxidation, resulting in the onset and progression of a variety of chronic diseases [42, 161, 162]. The area of hormetic responses triggered by nutritional supplements in enhancing or inhibiting endogenous redox antioxidant defense systems (e.g., Nrf2 pathway and GSH) is emerging as a promising preventive and therapeutic strategy in diseases associated with oxidative damage and inflammation, such as cancer [42].

Hormetic nutrients and their metabolites at low doses are considered chemopreventive agents, upregulating the Nrf2 pathway and downstream phase II detoxification vitagenes to induce cytoprotection, and can be used as useful dietary supplements in cancer chemoprevention to suppress oxidative stress and inflammation and avert proliferation in humans with genetic predisposition to develop cancer [167–169]. Mutations lead to constitutive activation and dysregulation of the Nrf2 pathway in cancer cells, resulting in drug resistance, immunological deficiencies, metabolic reprogramming, cancer growth, and metastasis. Overexpression of Nrf2 and the vitagene pathways play intrinsic roles in boosting the tumorigenic cascade, as well as prospective therapeutic interventions via inhibitors that stop the proliferation and chemoresistance of brain cancer cells by enhancing apoptosis [107].

Plant-derived secondary metabolites offer improved regulation over Nrf2/Keap1/ARE pathway with lower risk of acute and chronic complications. A myriad of nature-derived products have displayed promise regarding management of brain tumors. They represent huge potential in the management of brain tumors by regulating oxidative homeostasis. Many of them possess the ability to reach beyond BBB, thus preferentially being evaluated for CNS ailments.

### Apigenin

Apigenin, a flavone derived from natural sources possesses potential benefits in cancer prevention and therapy due to its ability to suppress cell growth in various cancer cells [170]. Apigenin can exert notable effects on human GSCs. It significantly decreases the self-renewal capacity, cell growth, clonogenicity, and invasiveness of GSCs at 25 µM [171]. Suppression of invasiveness in addition to self-renewal capacity of the stem cells, make it a unique mode of therapy. Additionally, it inhibits the activator and transducer of transcription 3, phosphorylation of c-Met, as well as its downstream effectors MAPK and Akt, leading to reduced expression levels of relevant markers. Apigenin also triggers the generation of ROS, initiating apoptosis through phosphorylation of p38 MAPK in malignant glioma cells at 50 µM [170, 172]. Interestingly, apigenin could induce apoptotic cascades selectively to glioblastoma cells, barring normal astrocytes, which strengthens the appeal of apigenin [172]. Moreover, apigenin treatment induces apoptosis to neuroblastoma cells, as well as inhibiting cell invasion by more than 90% [173, 174]. Apigenin can also improve the sensitivity of glioma cells to radiotherapy, thus playing somewhat an adjuvant role chiefly through repairing DNA damage in a HIF1α-mediated manner [175]. Additionally, it inhibits the phosphorylation of EGFR-mediated MAPK, Akt and mTOR signaling pathways, and decreases the expression of Bcl-xL which may transactivate the Nrf2 pathway in a dose-dependent manner [176].

Interestingly, both Nrf2/ARE pathway induction and inhibition have been reported with apigenin depending mainly on dose. It prevents DNA damage as well as provides antiapoptotic effect upto 20 µM while reduces cell viability at higher concentration (40 µM) [177]. Apigenin, on the other hand reduces neuronal damage through its antioxidative and antiapoptotic properties affecting the expression of p53 and Nrf2, as well as the transcription of their target genes, thus strengthening its candidature as a potential protective and therapeutic aid to do away with CNS ailments including cancer [177].

### Astaxanthin

Astaxanthin, a β-carotenoid derived from the green algae *Haematococcus pluvialis*, has been extensively studied for its ability to remove ROS [178, 179]. Astaxanthin yielded excellent results regarding antioxidant activity. Zhang et al. [180] used oxygen glucose deprived cells to assess antioxidant activity. Astaxanthin pretreatment improved cell viability (peaking at 20 µM), reduced ROS and membrane oxidation levels, and increased SOD activity. On the other hand, it enhanced mitochondrial membrane potential and HO-1 and Nrf2 expression. Further, co-treatment with a PI3K inhibitor reversed the enhanced expression of Nrf2 and HO-1, but a GSK3 inhibitor promoted Nrf2 translocation. These findings imply that astaxanthin protects against oxygen glucose deprivation via the PI3K/GSK-3/Nrf2 pathway [180]. Interestingly, cell viability decreases from peak with concentrations more than 20 µM/L, which might be attributed to a dose-dependent hormetic response, leading to pro-oxidant effects. Utilizing the same cell line, the protective effect of astaxanthin on mitochondrial function and antioxidant capacity in cells exposed to glutamate-mediated excitotoxicity was investigated. Pretreatment with 20 µM astaxanthin reduced MDA, protein carbonylation, and 8-oxo-2′-deoxyguanosine levels, indicating antioxidant action. In the mitochondria, astaxanthin reduces free radical generation. However, siRNA knockdown of Nrf2 prevents the effect of astaxanthin on HO-1 activity [181]. Later, neuroblastoma cells were stressed with H_2_O_2_, causing redox impairment and mitochondrial malfunction. Pretreatment with astaxanthin produced outcomes comparable to those above. In contrast, astaxanthin boosted HO-1 and Nrf2 activity via a mechanism related with the PI3K/Akt signalling pathway [182].

In animal models, pretreatment with astaxanthin (100 mg/kg) has improved neurological dysfunction caused by acute cerebral infarction. Astaxanthin increased the levels of antioxidant enzymes CAT, SOD, and GPx while decreasing lipid peroxidation. Astaxanthin elevated the expression of HO-1 and nuclear Nrf2, while decreasing the expression of cytosolic Nrf2 [183]. Similarly, in another investigation, astaxanthin increased neuronal function [184]. Astaxanthin raised the levels of HO-1 and Nrf2 proteins as well as improved nuclear import of Nrf2. Astaxanthin also confers protective effects against neurotoxicity at 60 mg/kg dose. Astaxanthin can even restore the levels of PI3K p110, PI3K p85, p-AKT, Nrf2, HO-1, NQO1, and GCLM [185]. The antioxidant activity of astaxanthin derived from *H. pluvialis* was assessed in rat model of non-arteritic anterior ischaemic optic neuropathy. The results revealed that both pre- and post-injury treatment with astaxanthin with dosage of 100 mg/kg daily for 7 days raised the levels of SOD, Nrf2, and p62 relative to the control rats [186].

Astaxanthin at 50 and 100 µM concentration dramatically reduced cell migration and invasion in glioblastoma cells [187]. The metastasis-reducing impact of astaxanthin is coupled with a dose-dependent reduction in MMP-2 and MMP-9 expressions. This finding revealed that astaxanthin could impart anti-migration and invasion actions against human glioblastoma cells and may be useful in the prevention of glioblastoma metastases [187]. Interestingly, astaxanthin at low concentrations protected glioma cells from UV-induced DNA damage, heavy metal and heat-induced protein misfolding, and protein aggregation. Long-term treatment in glioma cells resulted in physiological differentiation into astrocytes. These behaviors were supported by enhanced expression of proteins that regulate cell proliferation, DNA damage repair mechanisms, and glial differentiation, implying that they have the ability to prevent and treat stress, protein aggregation, and age-related diseases [188]. Unlike many other compounds, astaxanthin does not imply an anomalous list of findings, and result of Nrf2 upregulation has been clearly implicated in chemopreventive activity of astaxanthin.

### Baicalin

The search for new drug molecules to combat cancer has led to the discovery of baicalin, a flavonoidal compound found in the dry root of *Scutellaria baicalensis*. Baicalin exhibits great potential in the field of cancer management by triggering apoptosis, suppressing miRs, influencing the expression of proteins regulating cell death, enhancing the functions of Bax and p53, and increasing the levels of cyclin-dependent kinase inhibitors [189]. In relation to the management of glioma/glioblastoma, baicalin can effectively impede the proliferation and movement of brain cancer cells in a dose-dependent manner upto 300 µM concentration [190]. Baicalein, the aglycone of baicalin protects glial cells from oxidative stress and associated damage through regulation of the Nrf2 signaling pathway [191]. Interestingly, the cytoprotective functions of baicalein could be attenuated by transient transfection with Nrf2-specific siRNA [191]. Clearly, baicalein elevates cellular antioxidant defense via inhibition of ROS generation and activation of the Nrf2 signaling cascade. Additionally, the extracts derived from *Scutellaria baicalensis* have exhibited promise as anticancer agents for glioblastoma multiforme [192]. Baicalin effectively hinders the build-up of intracellular ROS, resulting in a significant restoration of mitochondrial membrane potential. This restoration was partially achieved by activating Nrf2 signaling and inducing HO-1 and TrxR1 [191, 193].

Bioavailability of baicalin is only around 2%. Therefore, the encapsulation of baicalin in nanoparticles has been anticipated to improve its bioavailability and facilitate its internalization in cancer cells. Interestingly, baicalin offers great potential in the field of cancer therapeutics also by triggering apoptosis, suppressing miRs, influencing the expression of proteins regulating cell death, enhancing the functions of Bax and p53, and increasing the levels of cyclin-dependent kinase inhibitors when delivered embedded in lipid nanoparticles at the concentration of 13 ± 5 µg/ml [189]. These nanocapsules induce a halt in the cell cycle resulting in a significant increase in the expression of the P21 gene and a decrease in the expression of Nrf2, HO-1, and VEGF genes in glioblastoma cells. Importantly, they are able to surpass BBB and achieve higher concentration in brain compared to free baicalin [189].

### Carnosic acid

Carnosic acid, a chemical compound abundant in rosemary and sage, possesses anti-inflammatory, antioxidant, and antitumor properties [194–196]. It can counteract free radicals, even within the brain [197]. Targeting tumor metabolism with carnosic acid presents a potentially promising approach to address drug resistance and enhance tumor sensitization in cancer therapy. Carnosic acid notably increases the levels of Nrf2 protein in cells. Additionally, it facilitates the nuclear translocation of Nrf2. It promotes the movement of Nrf2 into the cell nucleus, which then triggers the activation of genes involved in antioxidant defense. Clearly, carnosic acid potentially inhibits ferroptosis by activating the Nrf2 pathway [198].

Pretreatment with carnosic acid (1 µM) enhances the synthesis of GSH by increasing the activity of γ-glutamylcysteine ligase (γ-GCL) in neuroblastoma cells [199]. Carnosic acid promotes mitochondrial protection in neuroblastoma cells by activating the PI3K/Akt/Nrf2/γ-GCL/GSH pathway in the presence of toxicants. In addition, carnosic acid, at 1 µM safeguards neuroblastoma cells against the detrimental effects of glutamate-induced mitochondrion-related redox impairment and bioenergetic decline [200]. This mitochondrial protection is achieved through its interaction with Nrf2, as demonstrated by the suppression of these protective effects upon the silencing of this protein using siRNA. Carnosic acid prevents disruption of mitochondrial membrane potential, and reduces oxidative stress markers in the mitochondrial membranes neuroblastoma cells [201]. The protective action of carnosic acid (1 µM) has further been evidenced by its ability to increase the levels of GSH in mitochondria facilitated through the activation of the PI3K/Akt/Nrf2 signaling pathway [201]. Carnosic acid bears the ability to bind to specific cysteine residues of the Keap1 protein, which ultimately leads to the inactivation of Keap1 function. This results in the stabilization of Nrf2 and the activation of its transcriptional pathway, facilitating the expression of cytoprotective genes [198]. Moreover, carnosic acid is responsible for induction of nerve growth factor in glioblastoma cells via Nrf2 pathway, showing a comparatively high output after treating with 50 µM carnosic acid for 24 h [202]. Carnosic acid, interestingly, increases the effectiveness of temozolomide in targeting glioma cells [203]. Not only can it enhance the inhibition of colony formation and cell migration caused by temozolamide, but also heightens the impact of temozolamide on cell cycle arrest and cellular apoptosis. It also stimulates cellular autophagy induced by temozolamide through the inhibition of PI3K/Akt, and downregulation of p62. Downregulation of PI3K/Akt may be responsible for regulation of Nrf2 pathway which may further transactivate ARE genes. Despite these findings, it is apparent that only a limited amount of research has been conducted on the treatment of glioma/glioblastoma by carnosic acid thus far, and additional research is required for the purpose of clinical translation.

### Corilagin

Corilagin, an ellagitannin, exhibits potential for a range of biological activities modulating the Nrf2 cascade. There exists a higher level of Nrf2 expression in glioma tissues compared to non-glioma counterparts [204] Consequently, scientists have hypothesized that corilagin might possibly affect the regulation of Nrf2 regarding apoptosis of glioma cells. Corilagin stimulation can lead to a decrease in Nrf2 expression of glioma cells. Corilagin at 100 µg/ml concentration may induce apoptosis and autophagy by downregulating Nrf2 expression [204]. At the same concentration, corilagin can impede the proliferation of glioblastoma cells and glioblastoma stem-like cells, causing a halt in the cell cycle [205]. Corilagin opposes the growth of both glioblastoma multiforme cells, and glioblastoma stem-like cells at concentration upto 100 µg/ml [206]. A low concentration of corilagin can trigger the expression of the p65 gene promoter whereas a higher concentration inhibits this expression with a hormetic response. Clearly, dose plays a key role regarding the effect exerted by corilagin. Corilagin has also been effective against temozolamide-resistant glioma cells igniting a new hope regarding therapeutics of resistant-brain tumors. When used alongside temozolamide on glioma cells, a greater level of proapototic and antiproliferative effects has been observed [207]. Thus, studies have strongly supported the notion that corilagin could be considered as a potential drug for the treatment of glioma.

### Corosolic acid

Corosolic acid, obtained from *Actinidia chinensis* is widely used as a food supplement worldwide. It exerts anticancer effects by blocking transformation and epigenetic restoration of Nrf2 expression. Corosolic acid induces mRNA and protein expression of Nrf2 and HO-1 [208]. Moreover, corosolic acid reduces methylation of first 5 CpG sites of the Nrf2 promoter. It further attenuates protein expression of HDACs to halt tumorigenesis. On the other hand, it is also known to induce apoptosis by inhibiting nuclear translocation of NF-κB subunit p65, and activation of IκBα in a dose-dependent manner and effects increase upto 80 µg/ml concentration [209]. In addition, corosolic acid also induces non-apoptotic cell death by upregulating lipid peroxidation [210]. Thus, killing of cancer cells could be achievable with this molecule. The compound is also attributed to inhibition of VEGFR2 kinase activity corresponding to subsequent downregulation of Src/FAK/cdc42 signaling axis [211]. In brain, corosolic acid demonstrates anti-inflammatory, antioxidant, and neuroprotective activities [212]. Corosolic acid has been observed to promote ubiquitin-mediated proteasome degradation during glioblastoma [213]. It also inhibits activation of JAK2/MEK/ERK pathway affecting invasiveness of glioblastoma cells, suggesting anti-metastatic activity of corosolic acid on glioblastoma cells.

### Crocin

Crocin is a water-soluble antioxidant compound found in a variety of plant species [214, 215]. Crocin can essentially decrease cytoplasmatic expression of Nrf2 while increasing nuclear expression of Nrf2 [216]. A study sought to investigate the effects of crocin and their methyl ester derivate dimethylcrocetin on glioblastoma and rhabdomyosarcoma cells in terms of cytotoxicity and gene expression, which are involved in proapoptotic and cell survival pathways [217]. Both compounds conferred cytotoxic effects on glioblastoma and rhabdomyosarcoma cell lines in a dose and time-dependent manner. They triggered apoptosis by upregulating Bax and BID while downregulating MYCN and Bcl2, SOD1, and GSTM1 [217]. The results indicate a somewhat mixed role of crocin against brain cancer which might be attributed to the double-edged nature of the Nrf2 cascade itself.

### Curcumin

Curcumin, a pigment found in turmeric, has long been used in traditional medicine possesses antioxidant, anti-inflammatory, and antiproliferative effects. Curcumin is able to induce cytotoxic effects to tumor cells by virtue of cell cycle arrest, apoptosis, autophagy, changes in gene expression, and disruption of molecular signaling cascades [218]. Curcumin can essentially activate Nrf2 and induce protective mechanisms against oxidative stress. Emerging evidence suggests that Nrf2 activation could potentially improve the survival rate in glioblastoma [219]. In addition, curcumin acts as DNA hypomethylation agent to restore the expression of Nrf2 via demethylation of promoter CpGs [220]. Thus, curcumin can confer chemopreventive effect, in part, via epigenetic modification of the Nrf2 gene leading subsequently to induction of the anti-oxidative stress cellular defense mechanism mediated by Nrf2. Curcumin, on the other hand, possesses inhibitory effects to glioma cells aided by miR-378 too [221]. This effect becomes evident at doses of 60–120 mg/kg of curcumin. Moreover, curcumin downregulates the proliferation of glioblastoma cells via inhibition of miR-21, a regulator of glioblastoma progression [222]. Clearly, effects of curcumin on the Nrf2-cascade depends on dose. The overexpression of EGFR, closely associated with different types of cancers including brain cancer is regulated by several molecular pathways, including PI3K/Akt-dependent pathway [223]. Curcumin leads to dose-dependent antiproliferative effects on glioblastoma cells by inhibiting the overexpression of EGFR upto 30 µM concentration [224]. Rapid metabolism and limited stability, have contributed to the low bioavailability of curcumin, but when it is delivered locally into the brain within nanoformulations, its penetration into the targeted brain nuclei has been greatly enhanced [225, 226]. However, it is not yet established whether curcumin-induced Nrf2 activation would lead to an overall increase in the survival of glioblastoma patients.

### Fucoxanthin

Fucoxanthin, a xanthophyll obtained from marine sources has protective effects by inducing strong antioxidant effects against inflammatory pathways [227]. It is a potent activator of Nrf2, working via Akt/GSK-3β/Fyn axis against oxidative damage [228]. Fucoxanthin acts by blocking free radicals, thus reducing ROS levels in the body. Influencing the Akt/Nrf2 pathway, it enhances expression levels of both glutathione synthatase and GSH, thereby indicating lower susceptibility of cells to tumorigenesis which is at par with the findings stated in Table 2 [229]. Fucoxanthin is known to exert protection from DNA damage, especially in glioma cells [188] Long term treatment further upregulates DNA damage repair mechanisms and cell proliferation hinting at the potential of fucoxanthin to protect from stress-related pathologies. Interestingly, in a later experiment fucoxanthin exhibited antitumorgenic potential also, to glioblastoma cells, however this study also shows increase in cell-viability of glioblastoma cells at low concentration (10 µM) of fucoxanthin [230]. Fucoxanthin also exerts neuroprotection via Nrf2/ARE pathway [231]. Fucoxanthin induces apoptosis to glioma cells via inhibiting PI3K/Akt/mToR cascade at high concentration upto 100 µM but increases cell viability at a lower concentration of 6.25 µM [232]. A clear inclination towards dose-dependent hormetic response is thus indicated. Fucoxanthin also reduces migration and invasion by inhibiting PI3K and p38, thus strengthening its candidature as a promising molecule in the war against brain tumors.

### Icariin

Icariin can regulate Nrf2 by modulating multiple Nrf2 upstream activators. It can activate the Nrf2/ARE cascade to exert preventive actions [233]. In this way, it can even protect from neurotoxicity [234]. It is associated with increase in expressions of Nrf2, HO-1 and NQO1 [235] Icariin attenuates release of proinflammatory factors in the microglia. Emerging evidence suggests that icariin confers the effects by virtue of Nrf2 and HO-1 activation [236]. On the contrary, in a dose and time-dependant manner, it can induce apoptosis in medulloblastoma cells [237]. Clearly, regulation of Nrf2 mediated by icariin could play a vital role in the management of brain disorders including cancer [238].

### Luteolin

Luteolin, which is an active component found in *G. tenuifolia*, has been extensively studied in cellular research and has demonstrated various beneficial properties such as its ability to combat tumors, reduce inflammation, act as an antioxidant, and scavenge harmful radicals. Luteolin-induced response mainly involves upregulation of Nrf2, which regulates anti-inflammatory actions. Luteolin upregulates Nrf2 by virtue of demethylating effects on Nrf2 promoter regions of DNA [239, 240]. On the contrary, in cancer cells, luteolin has decreased cell viability in a dose-dependent manner [239]. Luteolin is hypothesized to cause apoptosis through Nrf2-p53 interactions. It triggers apoptosis through mitochondrial pathway characterized by Bax upregulation and Bcl-2 downregulation in glioblastoma cells [241]. Luteolin has exhibited significant inhibitory effects on cellular growth and migration, as well as the ability to arrest the cell cycle and induce cell death in glioblastoma cells with increasing concentrations upto 52.5 µM [242]. Additionally, luteolin can enhance the effectiveness of anticancer drug, while only causing minimal harm to normal cells [242].

Luteolin possesses the remarkable ability to safeguard neural cells from oxidative stress through the activation of Nrf2. Luteolin effectively shields neural and glial cells from the harmful effects of N-methyl-4-phenyl-pyridinium, showcasing its potential as a neuroprotective agent, however 5µM was found to be the highest non-toxic condition, above which cytotoxicity ensued [243]. This protection is intrinsically linked to the activation of Nrf2, as the suppression of Nrf2 completely nullifies the safeguarding effects of luteolin. Intriguingly, the neuroprotective impact of luteolin is compromised when the activation of ERK1/2 is inhibited. Thus, luteolin may potentially exert a protective effect on normal brain cells, safeguarding them against tumorigenesis.

The combined use of luteolin and other chemopreventive agents exhibits great potential as a therapy to prevent cell migration and invasion, and induce apoptosis in glioblastoma cells. Combination of luteolin and another flavonoid, silibinin, has been more effective in treating human glioblastoma cells and GSCs compared to single agents [244]. The combination effectively blocks angiogenesis and survival pathways, leading to the initiation of apoptosis. Ultimately, the inhibition of PKCα, XIAP, and iNOS resulted in the activation of both extrinsic and intrinsic pathways of apoptosis. Clearly, the findings suggest that extensive studies are required to determine the specific and dose-dependent activities and mechanisms of modulation of Nrf2/Keap1/ARE pathway to optimize its therapeutic attribute against brain cancer. To address the issue of comparatively lower bioavailability of luteolin aring from its hydrophobicity may be addressed by formulating it into nano-carriers [241].

### Lycopene

Lycopene, a carotenoid compound is abundantly available in our daily diet. Interestingly, it can be converted to β-carotene, the precursor of vitamin A by the enzyme lycopene-β-cyclase. Lycopene downregulates Nrf2, HO-1 and NQO1 while upregulating Keap1 [245]. However, some studies suggest that nuclear Nrf2 is uplifted by the action of lycopene [246, 247]. At 50 µM, lycopene enhances the production of Nrf2 and genes controlled by it, reduces ROS levels, and maintains the potential of the mitochondrial membrane [248]. Lycopene can effectively attenuate oxidative stress and inflammation by regulating Nrf2//NF-κB balance [245]. It plays vital role to maintain neuronal balance via regulating Nrf2/NF-κB pathway to minimize oxidative damage [249]. Within CNS, lycopene can be attributed to alleviation of oxidative stress via modulation of PI3K/Akt/Nrf2 cascade [250]. Moreover, dietary lycopene potentially exerts therapeutic benefit in high-grade gliomas [251].

### Pelargonidine

Pelargonidin is a plant anthocyanindin that exhibit antioxidant and anti-inflammatory activities against cancer cells [252]. Pelargonidin induces overexpression of the Nrf2/HO-1-signaling pathway to protect from oxidative stress, in addition to upregulating detoxifying enzymes via Nrf2/ Keap1 pathway at 50 µM [253, 254]. It also suppresses ROS-NLRP3-IL-1β axis via activating the Nrf2 signaling cascade [255]. Pelargonidin influences cells against neoplastic transformation by virtue of activation of Nrf2 pathway. It exerts cytoprotective activity by activating ARE-luciferase, and reducing protein levels of genes encoding methyltransferases and HDACs. Pelargonidin also decreases the DNA methylation in the Nrf2 promoter region, and increases expression of downstream target genes of Nrf2 e.g. NQO1 and HO-1 at concentrations upto 50 µM [256]. In glioma model, pelargonidine has been reported to deploy its activities via inhibition of phosphorylation of Akt, PI3K and mToR, and downregulation of VEGF [257].

### Quercetin

Quercetin is among the most abundant flavonoids of human diet. Quercetin is directly associated with upregulation of both nuclear and cytoplasmic Nrf2 [258, 259]. It promotes the expression of HO-1, NQO1, PI3K and SIRT1 as well as enhances activities of SOD, CAT, GPx and GST [260, 261]. Thus, quercetin and derivatives can provide protection from oxidative damage. Quercetin might be involved in activation of TrKB, which, in turn upregulates activation of Akt, an activator of Nrf2 [262]. Improved expression profile of glyoxalase-1 results from upregulation in nuclear interaction between Nrf2 and ARE [263]. Additionally, quercetin elevates the efficacy of chemotherapy and radiotherapy in case of brain tumors by improving the sensitivity of cancer cells to the treatments [264]. Quercetin can potentially regulate antioxidant levels in brain by modulating the Nrf2, Keap1 and associated pathways [265]. The concern regarding low bioavailability of quercetin may be addressed by designing suitable nano-scale delivery systems.

### Resveratrol

Resveratrol, a naturally occurring stilbene polyphenol exhibits a plethora of pharmacological attributes. It exerts the effects chiefly through epigenetic modification of the Nrf2 signaling cascade. Resveratrol is capable of elevating the methylation status of Nfe2l2 gene while lowering that of Keap1, resulting in decreased Nrf2 expression and activity [266]. Resveratrol can successfully cross BBB, making it a potential prophylactic and/or therapeutic agent against CNS disorders including cancer [267]. A growing body of evidence indicates that it can check carcinogenesis by interfering with initiation, proliferation, invasion, and metastasis processes [268]. Resveratrol simultaneously displays chemotherapeutic effects on cancer cells through a prooxidant mechanism promoting ROS production, induces endoplasmic reticulum stress, apoptosis, and cell cycle arrest in a dose- dependent manner decreasing cell viability with increase in concentration upto 10 µM [269, 270]. Emerging evidence suggests that resveratrol inhibits oncogenic miRs, e.g. miR-19, miR-21, and miR-30a-5p, subsequently leading to suppression of their target genes [15].

Interestingly, resveratrol enhances ROS level within cancer cells through a prooxidant mechanism. It induces a mitochondria-mediated imbalance regarding endogenous antioxidants resulting in an increased accumulation of ROS and lipid peroxides in cancer cells. Increased accumulation of ROS and lipid peroxide in turn induces oxidative stress in glioma cells and endorses apoptosis at 10 µM resveratrol concentration [271, 272]. Thus, resveratrol can serve as a potential chemotherapeutic agent too to treat brain tumors. In few cases, resveratrol can serve as a chemosensitizing agent in glioblastoma by virtue of additive prooxidant effects with another drug, amplifying ROS production, AMPK activation and mTOR inhibition [272, 273]. However, resveratrol exhibits poor water solubility, and bioavailability, raising concerns regarding its therapeutic efficacy. Fabrication of nano-scale cargos loaded with resveratrol might aid in overcoming the poor pharmacokinetic attributes in brain tumor management [274–276]. All these findings might be relevant in chemotherapeutic potential of resveratrol, however integrating research is required to tie the threads to completely explore efficacy of resveratrol against brain cancer.

### Sulphoraphane

Sulforaphane, an isothiocyanate derived from glucoraphanin found in large amounts in *Brassica* plants, has been extensively studied for its potential chemopreventive activity in recent decades [277]. Research indicates that sulforaphane imparts pleiotropic effects on cancer, affecting it at many stages from formation to progression [278]. Its chemopreventive ability stems from the capacity to activate the Nrf2 transcription factor, which in turn activates phase II detoxifying enzymes [279, 280]. The significance of this role arises from the low concentration of sulforaphane required to activate Nrf2 target genes [281]. Sulforaphane (0.2 µM) imparts stronger inducer activity on NQO1, a key Nrf2-activated enzyme, than many other phytochemicals including carotenoids [280, 282]. Furthermore, sulforaphane can cross the BBB and accumulate in the CNS [283]. As a result, using a pleiotropic medication that affects distinct cancer cell properties could be a viable method to fight glioblastoma [284]. Sulforaphane, at higher concentrations (> 10 µM) leads to death of glioblastoma cells, increasing their ROS levels [285]. However, sulforaphane may also protect normal cells from oxidative damage dose-dependantly [286, 287]. These paradoxical sulforaphane actions are linked to cancer cells’ intrinsic high levels of ROS, which may help to magnify the death signal caused by anticancer agents; in contrast, this does not occur in normal cells [288]. Sulforaphane also leads to apoptosis in CD133-positive glioma stem cells and dramatically inhibits the survival of CD133-positive and SOX2-expressing glioblastoma spheroids derived from glioblastoma cell lines [284]. The same study has inferred that oral intake of sulforaphane (100 mg/kg/day) reduces tumor growth and increases cell death in ectopic GBM10 xenografts. It is worth noting that sulforaphane may significantly decrease tumor growth in cancer xenografts, i.e. severe combined immunodeficiency mice implanted with GBM8401 cells [289].

### Tanshinone IIA

Tanshinone IIA, derived from *Salvia miltiorrhiza* has been linked to epigenetic activation of Nrf2 [290]. The robust stimulation of Nfe2l2 mRNA and Nrf2 protein levels by tanshinone IIA has been linked to hypomethylation of the Nfe2l2 promoter. It exerts pharmacological actions mainly via activating Nrf2/ARE cascade, and blocking NF-kB signaling pathway. Moreover, being lipophilic, it poses less problems regarding permeability. Again, tanshinone IIA can block the progression of carcinoma by destroying tumor cells, triggering apoptosis [291]. It inhibits cell proliferation, migration, and invasion of glioma cells chiefly through regulating mIR-16-5p in a dose-dependent manner [292]. Interestingly, tanshinone IIA plays crucial role to prevent alterations of BBB permeability during diseased state, thus making it an attractive agent for added protection [293]. It also exerts neuroprotective effect by regulating Nrf2/HO-1 pathway [294].

### Ursolic acid

Ursolic acid is a pentacyclic triterpenoid that activates the Nrf2 pathway by demethylating the Nfe2l2 promoter [295]. However, the same study found that a concentration of ursolic acid higher than 2.5 µM has cytotoxic potential thus confirming a hormetic dose response [295]. Ursolic acid also increases the expression of the protein methyltransferase SETD7. Moreover, it enriches H3K4me1 at the Nfe2l2 promoter, resulting in upregulation of Nrf2 signaling [296]. As a whole, ursolic acid activates the Nrf2/ARE pathway and reduce oxidative stress; however, a recent experiment with ursolic acid nanoparticles displays an antitumor effect of ursolic acid on glioblastoma at 20 mg/kg alongside promising penetration across BBB [297]. This is further supported by synergistic anticancer effects of sorafenib and ursolic acid, suggesting that the depletion of cancer cells can be attributed to selective apoptosis and ferroptosis considering the regulatory effect on the Nrf2 pathway [298]. The contradicting effect of ursolic acid on Nrf2 cascade indicates the hormetic dose response exerted by ursolic acid.

## Nature-derived Nrf2 modulators: an outlook towards clinical translation

Many studies based on Nrf2 cascade modulators express immense promise in both chemopreventive and chemotherapeutic axis against brain cancer (Table 5). However, the novel approach has not yet been extensively analyzed through translational clinical research, leaving only few clinical studies based on brain cancer models to summarize. Among the clinically tested plant-derived Nrf2-regulators, sulforaphane, an isothiocyanate compound obtained from cruciferous vegetables has been a leading example [131]. The ability of sulforaphane to permeate BBB has been one of the main point of interest apart from its role in upregulating Nrf2 and HO-1. This compound can also ameliorate several stress-mediated neurodegenerative diseases [299, 300]. Sulforaphane has proven detrimental against brain cancer cells while protecting normal cells [285, 301]. Continuing the search, sulforaphane has been subjected to several anticancer clinical trials [302, 303], and thus has the potential to be studied clinically against brain cancer as well. Curcumin, a linear diarylheptanoid found in turmeric, is another naturally-occuring substance which alters Cys-151 in Keap1 while also scavenging ROS [304]. Curcumin reduces the detrimental effects of carcinogens by activating Nrf2 [305, 306]. On the other hand, curcumin treatment in glioblastoma cells reduces malignant features, enhances chemotherapeutic efficacy, and increases apoptosis in cancer cells [307–310]. The dose-dependent variable response makes it an attractive candidate for clinical translation. Curcumin is presently subjected to an ongoing phase III anticancer clinical trial of prostate cancer (NCT02064673) whereas other clinical trials for diseases of CNS have yielded promising results [311, 312]. Combining these with the inherent ability of curcumin to cross the BBB, it becomes suitable candidate for management of brain cancer. Resveratrol exerts anticancer properties through depleting Nrf2 cascade and enhanced intrinsic ROS generation [266, 268]. Additionally, it penetrates BBB and has been found to deliver chemotherapeutic effect in various preclinical studies against brain cancer [313–315]. Due to these promising results in preclinical evaluations, resveratrol has been subjected to a phase I clinical trial against colon cancer and at the used dose, showed potential in chemoprevention [316]. None of these studies however specifically encompasses the domain of brain cancer therefore the huge gap in translational studies of Nrf2 modulators on brain cancer is evident, even though the opportunity for exploration is heavily backed by pre-clinical findings. In conclusion, it seems that Nrf2 inhibitors should be given at advanced stages of cancer for chemotherapy and Nrf2 activators should be used to prevent cancer recurrence/post-chemotherapy/prophylactic manner. However, it is quite difficult to pinpoint a specific time-frame due to scarcity of positive clinical evidence regarding brain tumors.

**Table 5:** Promising plant secondary metabolites acting on brain cancers via modulating Nrf2 signaling cascade

Sl No.	Secondary metabolites	Plant sources	Modulation of Nrf2-associated signaling cascade	Diseases studied	References
1	Apigenin	*Matricaria chamomilla*, *Apium graveolens*	Induction of apoptosis to tumor cells, Protective effect to healthy neurons	Glioma, neuroblastoma	[170, 174, 175]
2	Astaxanthin	*Haematococcus pluvialis*	Upregulation of nuclear Nrf2, dose-dependant reduction in expression of MMPs	Glioblastoma	[187]
3	Baicalin	*Scutellaria baicalensis*	Reduced intracellular ROS, induction of HO-1	Glioma	[191]
4	Carnosic acid	*Rosmarinus officinalis*	Enhanced nuclear translocation of Nrf2, enhanced GSH, inactivation of Keap1 function	Glioblastoma, neuroblastoma	[199, 200, 202, 203]
5	Corilagin	*Caesalpinia coriaria*, *Punica granatum*	Induction of apoptosis and autophagy, antiproliferative effect	Glioblastoma	[204, 206]
6	Corosilic acid	*Actinidia chinensis*, *Actinidia* valvata, Lagerstromeia sp.	Inhibition of nuclear translocation of NF-κB subunit p65, activation of IκBα, promotion of ubiquitin-mediated proteasome degradation	Glioblastoma	[213]
7	Crocin	*Crocus sativus*	Induction of apoptosis	Glioblastoma	[217]
8	Curcumin	*Curcuma longa*	Decreased methylation of Nfe2l2 promoter, reduced cell proliferation	Glioblastoma	[219]
9	Fucoxanthin	*Sargasso* sp., *Luminaria* sp.,*Phaeodactylum tricornutum*	Reduced ROS, enhanced GSH, protection against DNA damage	Glioma	[188, 230]
10	Icariin	*Epimedium brevicornum*	Activation of Nrf2 and HO-1, dose-dependant induction of apoptosis	Medulloblastoma	[235]
11	Luteolin	*Glossogyne tenuifolia*	Triggering of apoptosis in tumor cells, neuroprotection	Glioblastoma	[241, 244]
12	Lycopene	*Solanum lycopersicum*	Regulation of Nrf2//NF-κB balance	Glioma	[249, 251]
13	Pelargonidin	*Vaccinium* sp., *Geranium* sp.	Inhibition of phosphorylation of Akt, PI3K and mToR, and downregulation of VEGF	Glioma	[257]
14	Quercetin	*Citrus sp.*, *Malus domestica*	Regulation of brain antioxidant levels	Different types of brain tumors	[264]
15	Resveratrol	*Polygonum cuspidatum*	Decreased methylation of the Nfe2l2 promoter, reduced drug resistance	Glioblastoma	[270, 274]
16	Sulforaphane	*Brassica sp.*	Activation of Nrf2 at low concentration, increased ROS in cancer cells at high concentration	Glioblastoma	[280, 282, 285]
17	Tanshinone IIA	*Salvia miltiorrhiza*	Decreased methylated CpGs in Nfe2l2 promoter, decreased proliferation of glioma cells	Glioma	[292]
18	Ursolic acid	*Clinopodium revolutum*, *Malus* sp.	Demethylation of Nfe212 promoter	Glioblastoma	[297]

## Discussions and perspectives

Overall, the role of Nrf2 activation in cancer is paradoxical calling for further explorations. For the prevention of diseases like cancer whereby oxidative and inflammatory stress majorly contribute to pathogenesis, upregulating Nrf2 activity is an effective approach [317]. Interestingly, studies in the recent past have unveiled that overactivation of Nrf2 promotes the growth and proliferation of cancer cells, blocks cell apoptosis, and strengthens the self- renewal capability of cancer stem cells. Furthermore, overactivation of Nrf2 cascade augments the chemoresistance and radioresistance of cancer cells [318]. Hence, it is quite reasonable to consider that blocking the Nrf2 activity in fully malignant cells may be a logical approach for cancer eradication. It is evident that Nrf2/Keap1/ARE pathway plays a major role in cancer prevention by anti-inflammatory effects and reducing oxidative stress to reduce DNA damage leading to tumorigenesis. Chemoprevention in brain cancer by activation of Nrf2/Keap1/ARE pathway has come up as a very attractive approach, and therapeutic exploitation on this approach can lead to groundbreaking discoveries in the treatment and prevention of brain tumors. On the contrary, recent research indicate that inhibition of Nrf2/Keap1/ARE pathway can lead to anticancer activities [155]. However, agents used in treatment of brain cancer that downregulates Nrf2 can also prove to be neurodegenerative as due to enhanced oxidative stress, they might trigger neuronal degeneration [319]. Hence, new strategies and treatment methods are required to alleviate such adverse events.

Research has provided a large number of Nrf2 activators and blockers from the pool of plant-derived molecules that regulate Nrf2 at different levels and exert the anticancer effects [15, 320]. Many of the nature-derived small molecules exhibit promises in Nrf2 modulation, which still requires further investigations for optimization and clinical translation. Many cases, they even improve the sensitivity of tumor cells to radiotherapy and chemotherapy (with another drug). An ideal regulator for clinical application needs not only potency, efficiency and specificity but also low toxicity, high bioavailability and favorable pharmacokinetic profile. Many of the nature-derived small molecules under discussion can inherently cross BBB, making them potent candidates for management of brain cancers. Formulation development might aid in solving problems regarding targeting, specificity, and pharmacokinetics. An alternative strategy would be to explore indirect methods such as the inhibition of upstream miRNAs and/or protein kinases, along with directly targeting Nrf2. For the most part, to achieve consistent clinical outcomes with regard to the management of brain cancers by regulating Nrf2-associated signaling cascades, rigorous mechanistic studies are the need of the hour with respect to the types, forms, and stages of brain tumors.

## Conclusion

It is becoming evident with time that the transcription factor Nrf2 can suppress tumorigenesis as well as protect tumors against oxidative stress, and different forms of cancer may exhibit dysregulation of the signaling pathway at different levels, contexts, and downstream mechanisms. Through the production of antioxidant target genes, Nrf2 pathway hyperactivation can assist malignant cells in overcoming oxidative stress, hence increasing cell survival and proliferation. Additionally, Nrf2 can be crucial in the development of chemoresistance by minimizing the oxidative stress induced by drugs/therapeutic agents inside cancer cells and thus, shielding the cells from destruction. It is evident that the therapeutic utility of regulating Nrf2 depends on majorly on the type of cancer, disease stage, dose, and on other factors that can contribute to Nrf2 activation. An ideal balance between the disease-preventing and the disease-promoting effects of Nrf2 can provide useful benefit for cancer patients in future. The key line between the pro- and anti- oxidant effects is the decider for the use of an exogenous agent in terms of achieving the clinical success against brain cancer. Multiple advances in the understanding of Nrf2 signaling in brain cancer have emerged, but still a great deal of investigations remains to be performed to determine the specific mechanistic and functional underpinnings of the dual role of Nrf2/Keap1/ARE cascade.

## Data Availability

No datasets were generated or analysed during the current study.

## Abbreviations

β-TrCP: Beta-transducin repeat-containing protein
γ-GCL: γ-glutamylcysteine ligase
ACOX: Acyl-CoA oxidase
ARE: Antioxidant response element
ATRA: All-trans-retinoic acid
BACH1: BTB and CNC homology 1
BBB: Blood-brain barrier
CAT: Catalase
CNS: Central nervous system
EGFR: Epidermal growth factor receptor
EGFRvIII: epidermal growth factor receptor variant III
EMT: Epithelial-mesenchymal transition
ER: Endoplasmic reticulum
G6PD: Glucose-6-phosphate dehydrogenase
GPx: Glutathione peroxidase
GSC: Glioblastoma stem cell
GSH: Reduced glutathione
GST: Glutathione-S-transferase
HDAC: Histone deacetylase
HIF: Hypoxia-inducible factor
hMTH1: Human MutT homolog protein 1
IGF: Insulin-like growth factor
Keap1: Kelch-like ECH-associating protein 1
Maf: Musculoaponeurotic fibrosarcoma
miRNA: microRNA
Neh: Nrf2 ECH homology
Nrf2: Nuclear factor erythroid 2–related factor 2
PDI: Protein disulfide isomerase
PGD: Phosphogluconate dehydrogenase
PPP: Pentose phosphate pathway
PTEN: Phosphatase and tensin homolog
RNS: Reactive nitrogen species
ROS: Reactive oxygen species
Sirt1: Sirturin 1
SQSTM: Sequestosome
SOD: Superoxide dismutase
TALDO: Transaldolase
TCA: Tricarboxylic acid
TGF-β: Transforming growth factor beta
TKT: Transketolase
VEGF: Vascular endothelial growth factor
VEGFR: Vascular endothelial growth receptor
